# L-Proline-Mediated Modulation of Astringency in Black Chokeberry Puree: Molecular Interactions, Process Optimization, and Quality Preservation

**DOI:** 10.3390/foods15132388

**Published:** 2026-07-04

**Authors:** Wanru Zhao, Shiwei Yuan, Xin Wang, Jianyi Wang, Li Sheng, Yongqi Yin, Kai Song

**Affiliations:** 1 School of Life Science, Changchun Normal University, Changchun 130032, China; zhaowanrumei@163.com (W.Z.); ccsfysw00@163.com (S.Y.); wangxinresearch@163.com (X.W.); wjyhyjzxy@163.com (J.W.); 2 Jilin Qiuzhiyuan Ecological Technology Co., Ltd., Lishu County, Siping 136000, China; qzy_shengli@163.com; 3 College of Food Science and Engineering, Yangzhou University, Yangzhou 210095, China; yqyin@yzu.edu.cn; 4 Institute of Innovation Science and Technology, Changchun Normal University, Changchun 130032, China

**Keywords:** black chokeberry, L-proline, modulation-based deastringency, anthocyanin retention, in vitro digestive stability

## Abstract

*Aronia melanocarpa* puree is rich in anthocyanins and proanthocyanidins, but its pronounced tannin-derived astringency limits product acceptance. This study developed a non-removal astringency-modulation strategy using food-grade L-proline and evaluated its molecular basis, processing window, and quality effects. Ultraviolet–visible (UV–Vis) and Fourier-transform infrared (FT-IR) spectroscopic analyses suggested that L-proline altered the local microenvironment of procyanidin B2 (PC-B2) through hydrogen bonding, hydrophobic association, and molecular packing rearrangement, without evident disruption of the PC-B2 aromatic skeleton. In a PC-B2–bovine serum albumin model, an appropriate L-proline level reduced the protein precipitation rate from 45.3% to 31.2% and increased soluble phenolic retention, suggesting weakened polyphenol–protein precipitation. The strategy was then optimized in puree using machine learning-assisted multi-response analysis and Box–Behnken validation. The recommended condition was 150 mg/100 mL L-proline at 40 °C for 60 min, yielding a deastringency rate of 36.13%, with anthocyanin and vitamin C retention rates of 88.80% and 55.56%, respectively. The optimized treatment maintained red color, colloidal dispersion, and shear-thinning behavior; increased the anthocyanin digestion retention index from 50.0% to 87.4%; and improved overall sensory acceptance from 4.17 to 8.17. These findings support L-proline-mediated microenvironmental modulation as a mild processing approach for high-tannin cloudy berry products.

## 1. Introduction

Black chokeberry (*Aronia melanocarpa*) is a typical polyphenol-rich small berry containing abundant anthocyanins, proanthocyanidins, phenolic acids, and flavonoids, and therefore has considerable value for functional food development [1,2]. However, its high tannin content also imparts marked astringency and an oral puckering sensation to the fruit and its puree products, thereby limiting fresh consumption, consumer acceptance of processed products, and high-value utilization [3,4]. For cloudy puree products, quality improvement requires a balance between reducing the negative sensory impact of astringency and preserving polyphenols, pigments and the colloidal matrix. Therefore, establishing a mild processing strategy that balances deastringency efficiency with nutritional quality preservation is a key issue in the development of black chokeberry products [5,6].

Astringency is mainly associated with non-covalent interactions between condensed tannins, especially proanthocyanidins, and salivary proteins [7]. The polyhydroxyl groups and aromatic rings of proanthocyanidins can bind proteins at multiple sites through hydrogen bonding, hydrophobic interactions, and aromatic stacking, thereby inducing protein cross-linking, aggregation, and precipitation. Salivary proline-rich proteins (PRPs), owing to their relatively open conformations and abundant proline residues, are considered major target proteins for polyphenol binding [8,9]. Once polyphenol–protein complexes are formed, the lubricating layer of the oral mucosa is weakened and the oral friction state is altered, generating sensory perceptions such as dryness, roughness, and puckering. Accordingly, reducing the effective activity of PC-B2 in inducing protein precipitation represents a direct entry point for improving the astringency of black chokeberry puree [10,11].

Current food deastringency methods mainly include adsorption clarification, protein precipitation, enzymatic treatment, fermentation, and thermal processing [12,13]. For high-polyphenol cloudy systems such as black chokeberry puree, these approaches have limitations. Adsorption or clarification treatments are generally removal-based and may lead to losses of active compounds such as anthocyanins and total phenols while reducing tannins [14,15,16]. Strong thermal treatment may reduce some astringency but can accelerate vitamin C degradation and color deterioration [17,18]. Fermentation and enzymatic methods may alter the original flavor profile and increase process control complexity [19,20]. Thus, a deastringency strategy more suitable for cloudy puree should avoid extensive tannin removal and instead modulate the effective binding state of astringency-related polyphenols, weakening their protein-precipitating capacity while maintaining nutritional composition and colloidal stability [21,22].

Based on this rationale, food-grade L-proline was used in this study as a microenvironmental modulator of polyphenol molecules. L-proline is a natural free amino acid containing a pyrrolidine ring, an amino group, and a carboxyl group, which provides a structural basis for non-covalent association with polyphenols [23,24]. Unlike conventional deastringency strategies centered on precipitation, adsorption, or degradation, exogenous free L-proline is not intended primarily to remove proanthocyanidins. Instead, it may reduce the tendency of proanthocyanidins to form cross-linked precipitates with salivary proteins by competitively occupying some interaction sites, altering solvation states, or modulating local conformation [25,26]. Because this strategy does not rely on filtration or clarification, it is theoretically more suitable for integration into mild processing workflows for cloudy purees and composite beverages.

However, a real puree system is not a simple binary polyphenol–protein system; it contains proanthocyanidins, anthocyanins, pectin, organic acids, vitamin C, colloidal particles, and other components simultaneously [27,28,29]. The amount of L-proline added, processing temperature, and processing time not only affect deastringency efficiency but also influence anthocyanin retention, vitamin C stability, colloidal dispersion, and rheological properties [30,31]. Traditional response surface methodology (RSM) can be used for local process optimization, but its quadratic polynomial structure has limited capacity to describe complex threshold effects and nonlinear interactions [32,33]. Random forest (RF), back-propagation artificial neural network (BP-ANN), and interpretable machine learning methods can identify variable contributions and response trade-offs across a broader process space. Combining RSM with machine learning can create complementarity between locally verifiable optimum conditions and nonlinear multi-response interpretation, thereby improving the robustness and interpretability of process screening [34,35].

Therefore, this study used black chokeberry puree as the research object and performed multilevel validation of an L-proline-mediated, non-removal-based deastringency strategy. First, using PC-B2 and PC-B2–BSA model systems, UV-Vis spectroscopy, FT-IR spectroscopy, and protein precipitation assays were combined to clarify the regulatory effects of L-proline on the microenvironment of proanthocyanidin molecules and their protein-precipitating capacity. Second, the strategy was extended to the real puree system. Machine learning models were built using 125 five-level full-factorial observations, and SHAP interpretation, Pareto non-dominated sorting, and an overall desirability function were used to screen balanced multi-response processing conditions; a Box–Behnken RSM design was then applied to locally validate the region surrounding the recommended condition. Finally, the effect of the optimized treatment on integrated puree quality was evaluated in terms of color, particle size and zeta potential, rheological behavior, simulated in vitro digestive stability, and sensory quality. Through this stepwise study, we aimed to establish a mild processing approach that integrates astringency mitigation, nutritional preservation, and cloudy-system stability, thereby providing a theoretical basis and process reference for quality modulation of high-tannin berry purees and related composite beverages.

## 2. Materials and Methods

### 2.1. Materials and Reagents

Black chokeberry (*Aronia melanocarpa*) fruits were collected from the Tiexibulaomei Science and Technology Courtyard(Lishu County, Siping, China) in July 2025. Fruits with uniform maturity, without mechanical injury or visible disease or pest damage were selected, washed, drained, and stored at −20 °C until use. L-proline, PC-B2, bovine serum albumin (BSA), Folin–Ciocalteu reagent, 2,2-diphenyl-1-picrylhydrazyl (DPPH),2,2′-azino-bis (3-ethylbenzothiazoline-6-sulfonic acid) (ABTS),6-hydroxy-2,5,7,8-tetramethylchroman-2-carboxylic acid (Trolox), pepsin, pancreatin, and bile salts were purchased from Shanghai Macklin Biochemical Co., Ltd. (Shanghai, China) and Suzhou Grace Biotechnology Co., Ltd. (Suzhou, China). Unless otherwise specified, all reagents were of analytical grade or higher, and deionized water was used for all experiments.

### 2.2. Preparation of Black Chokeberry Puree

Thawed fruits were mixed with deionized water at a solid-to-liquid ratio of 1:5 (*m*/*v*) and homogenized with a high-speed tissue disintegrator (S10, Ningbo Sctentz Biotechnology Co., Ltd., Ningbo, China). The resulting puree was passed through a 20-mesh sieve to remove large peel and seed residues. Samples without L-proline addition or heat treatment were used as the original control (CK). Samples with L-proline addition and treated under the preset conditions were defined as the Pro-treated groups. At least three independent replicates were prepared for all treatments.

### 2.3. L-Proline Deastringency Treatment

An aliquot of 100 mL of black chokeberry puree was supplemented with 50, 100, 150, 200, or 250 mg of L-proline, corresponding to 0.5–2.5 g/L. After thorough stirring, the samples were placed in a thermostatic water bath (DK-8D, Shanghai Boxun Medical Biological Instrument Corp., Shanghai, China) and treated at 20–60 °C for 30–150 min. After treatment, the samples were immediately cooled to room temperature in an ice-water bath to terminate further thermal action. Each treatment included at least three independent replicates.

Single-factor experiments were conducted to evaluate the effects of L-proline dosage (0.5–2.5 g/L), processing temperature (20–60 °C), and processing time (30–150 min) on the deastringency rate and quality indices. On this basis, two complementary optimization modules were designed. The first was a five-level full-factorial experiment consisting of 125 runs, which was used for machine learning modeling and nonlinear-response interpretation across a broad process space (Section 2.9 and Section 2.10). The second was a three-factor, three-level Box–Behnken response surface design, which was used to establish a local quadratic polynomial model and validate response curvature, factor interactions, and model reliability around the machine learning-recommended conditions (Section 2.11).

### 2.4. Effect of L-Proline on the UV-Vis Absorption Spectra of the PC-B2-BSA System

BSA working solution (2.0 mg/mL), PC-B2 stock solution (1.0 mmol/L), and L-proline working solution (1.0 mmol/L) were prepared in 50 mmol/L PBS (pH 7.0). Two groups were established: the CK group (BSA-PC-B2) and the Pro group (BSA-L-proline-PC-B2). For each assay, a 3.0 mL aliquot of BSA working solution was used; 300 µL L-proline working solution was added to the Pro group, whereas an equal volume of PBS was added to the CK group. After the mixtures were equilibrated at 25, 35, or 45 °C for 10 min, 10 µL of PC-B2 stock solution was added in ten stepwise additions. After each addition, the mixture was mixed thoroughly and allowed to stand for 2 min. UV-Vis absorption spectra were recorded using a UV–Vis spectrophotometer (UV-58o0Pc, ShanghaiMetash Instruments Co., Ltd., Shanghai, China) across 270–450 nm, and changes in A_280_ were compared.

### 2.5. Fourier-Transform Infrared Spectroscopy

Mixed PC-B2:L-proline systems were prepared at a 1:2 molar ratio and freeze-dried. L-proline, PC-B2, and the freeze-dried using a freeze dryer (SCIENTZ-1oN/C. NingboScientz Biotechnology Co., Ltd., Ningbo, China) mixture were separately blended with dry KBr and pressed into pellets. FT-IR spectra were collected using a Fourier-transform infrared spectrometer (Nicolet iS50, Thermo Fisher Scientific, Waltham, MA, USA) over 4000–400 cm^−1^, with a resolution of 4 cm^−1^ and 32 scans. After baseline correction and smoothing, spectra were used to analyze the main characteristic peaks and changes in the functional group environment.

### 2.6. Proanthocyanidin–Protein Precipitation Inhibition Assay

A PC-B2-BSA model system was used to evaluate the inhibitory effect of L-proline on PC-B2-induced protein precipitation. Four groups were included: CK, Pro-L, Pro, and Pro-H. CK represented the PC-B2-BSA control system without L-proline addition, whereas Pro-L, Pro, and Pro-H corresponded to final L-proline concentrations of 0.167, 0.667, and 1.333 mg/mL, respectively. PC-B2 working solution (1.0 mL) was pre-incubated with L-proline working solution (1.0 mL) at 37 °C for 30 min. Then, 1.0 mL of BSA working solution was added, and the reaction was continued for another 30 min, followed by standing at room temperature for 10 min. A 200 µL aliquot of the reaction mixture was used to measure turbidity at 600 nm using a microplate reader (FlexA-200, Hangzhou Allsheng Instruments Co., Ltd., Hangzhou, China). The remaining reaction mixture was centrifuged using a refrigerated centrifuge (TGL-16M, Changsha Xiangyi Centrifuge Instrument Co., Ltd., Changsha, China) at 10,000× *g* and 4 °C for 15 min, and the precipitated protein content was determined using the BCA method. The calculations were as follows:

Protein precipitation rate (%) = (precipitated protein amount/initial total protein amount) × 100

Precipitation inhibition rate (%) = [1 − (precipitated protein amount in the Pro-treated group/precipitated protein amount in the PC-B2-BSA model group)] × 100

### 2.7. Determination of the Deastringency Rate Based on BSA Protein Precipitation Capacity

Each treated sample was centrifuged at 10,000× *g* and 4 °C for 15 min, and the supernatant was collected and appropriately diluted with PBS. A 1.0 mL aliquot of sample supernatant was mixed with 1.0 mL of BSA working solution (2.0 mg/mL), incubated at 37 °C for 30 min, and then allowed to stand at room temperature for 10 min. PBS was used instead of BSA to prepare the sample blank, thereby correcting for interference from endogenous precipitates. Precipitated protein content was determined using the BCA method, and the deastringency rate was calculated as follows:

Deastringency rate (%) = [1 − (effective protein precipitation amount in the treated group/effective protein precipitation amount in the untreated control group)] × 100

This index was used to characterize the relative reduction in the ability of astringency-related polyphenols in the sample to induce protein precipitation, and it was subsequently verified against sensory evaluation results.

### 2.8. Determination of Anthocyanins, Total Phenolics, Vitamin C, Flavonoids, and Antioxidant Activity

Total anthocyanin content (TAC) was determined using the pH differential method and the results were expressed as cyanidin-3-glucoside equivalents. The calculation was as follows:

TAC = (A × MW × DF × 1000)/(ε × l)

where A = [(A_520_ − A_700_) pH 1.0 − (A_520_ − A_700_) pH 4.5], MW = 449.2 g/mol, ε = 26,900 L/(mol·cm), l = 1 cm, and DF is the dilution factor. Anthocyanin retention rate (%) = (TAC in the treated group/TAC in the control group) × 100.

Total phenolic content was determined using the Folin–Ciocalteu method. Briefly, 10 µL of supernatant was mixed with 50 µL Folin–Ciocalteu reagent and incubated in the dark at room temperature for 3 min. Then, 50 µL of 10% sodium carbonate solution and 90 µL of distilled water were added. After mixing, the reaction was allowed to proceed at room temperature for 30 min, and the absorbance was measured at 760 nm.

Vitamin C content was determined by 2,6-dichloroindophenol titration. A 5.0 g sample was extracted with 50 mL of 2% oxalic acid solution and centrifuged at 10,000× *g* at 4 °C for 10 min. The extract was titrated with standardized 2,6-dichloroindophenol solution until a light-pink color persisted for 15 s. Vitamin C retention rate (%) = (content in the treated group/content in the control group) × 100.

Total flavonoid content was determined by the sodium nitrite–aluminum nitrate colorimetric method. An 80 µL aliquot of supernatant was mixed with 25 µL of 5% sodium nitrite solution and allowed to stand at room temperature for 6 min. Then, 50 µL of 10% aluminum nitrate solution was added and the mixture was incubated for another 6 min. Subsequently, 165 µL 4% sodium hydroxide solution was added, and the mixture was mixed and incubated at room temperature for 15 min. After centrifugation at 8000 rpm for 5 min, the absorbance of the supernatant was measured at 510 nm.

Antioxidant activity was determined using DPPH and ABTS radical-scavenging assays. For the DPPH assay, the test solution was mixed with an equal volume of 0.1 mmol/L DPPH ethanol solution, incubated in the dark at room temperature for 30 min, and the absorbance was measured at 517 nm. A blank was prepared using 80% methanol, and the radical-scavenging capacity was calculated and expressed as Trolox equivalents. For the ABTS assay, 7 mmol/L ABTS and 2.45 mmol/L potassium persulfate were allowed to react in the dark for 12 h to prepare the ABTS+ working solution, which was then diluted with absolute ethanol to an absorbance of 0.70 ± 0.02 at 734 nm. The test solution was mixed with the working solution, incubated in the dark at room temperature for 6 min, and the absorbance was measured at 734 nm. A blank was prepared using absolute ethanol, and the radical-scavenging capacity was calculated and expressed as Trolox equivalents.

### 2.9. Five-Level Full-Factorial Experimental Design

L-proline dosage (X1: 50–250 mg), processing temperature (X2: 20–60 °C), and processing time (X3: 30–150 min) were used as independent variables, each at five levels, to construct a 5 × 5 × 5 (125-run) full-factorial design. The response variables were deastringency rate (Y1), anthocyanin retention rate (Y2), and vitamin C retention rate (Y3). This dataset was used specifically for random forest (RF) and BP-ANN machine learning modeling and SHAP-based interpretability analysis (Section 2.10).

### 2.10. Random Forest and BP-ANN-Assisted Modeling

Random forest (RF) and BP artificial neural network (BP-ANN) models were developed to describe the nonlinear relationships between processing variables and response variables. The input variables were L-proline dosage, processing temperature, and processing time, and the output variables were deastringency rate, anthocyanin retention rate, and vitamin C retention rate. The machine learning dataset included 375 observations obtained from 125 full-factorial treatment combinations with three biological replicates for each combination.

The entire dataset was randomly divided into an 80% training subset and a 20% independent test subset using a fixed random seed (random_state = 42). The independent test subset was not used for model fitting and was used only for held-out evaluation of final prediction performance. To further assess model robustness and reduce dependence on a single data split, five-fold cross-validation was performed, consisting of five iterative rounds of training and validation. Model performance was evaluated using the coefficient of determination (R^2^), root mean square error (RMSE), and mean absolute error (MAE).

For RF, 500 decision trees were used, with no maximum depth limitation and a min_samples_leaf value of = 2. For BP-ANN, a 3-64-32-16-3 network structure with the tanh activation function was used, and the input variables were standardized before model training. SHAP analysis, Pareto non-dominated sorting, and an overall desirability function were then applied based on the RF model to interpret variable contributions and identify balanced multi-response processing conditions.

### 2.11. Box–Behnken Response Surface Design

To further validate the local response patterns around the machine learning-recommended conditions, a three-factor, three-level Box–Behnken design (BBD) was used to establish a quadratic polynomial response surface model. The variable ranges were processing time (A), 30–90 min; processing temperature (B), 30–50 °C; and L-proline dosage (C), 100–200 mg. The design included various factor-level combinations and five center-point replicates. The quadratic polynomial model was as follows:

Y = β0 + β1A + β2B + β3C + β12AB + β13AC + β23BC + β11A^2^ + β22B^2^ + β33C^2^

Analysis of variance was used to evaluate model significance, individual factor effects, and lack of fit. Response surface and contour plots were used to analyze local response patterns around the recommended process region.

### 2.12. Color Difference, Particle Size, and Zeta Potential Measurements

Color parameters L*, a*, and b* were measured using a colorimeter (NH310, Shenzhen Sanenshi Technology Co., Ltd.,Shenzhen, China), at three positions on each sample. The untreated control was used as the reference, and the total color difference was calculated as follows:

$$\Delta E\;=\;\sqrt{{\left(L^{*}-{L0}^{*}\right)}^{2}+{\left(a^{*}-{a0}^{*}\right)}^{2}+{\left(b^{*}-{b0}^{*}\right)}^{2}}$$

Particle size distribution and zeta potential were measured using a nanoparticle size and zeta potential analyzer (BeNano 180 Zeta, Bettersize Instruments Ltd., Dandong, China). Samples were diluted 100-fold with deionized water, and the average particle size, PDI, and zeta potential were measured at 25 °C. Each sample was measured in triplicate.

### 2.13. Rheological Properties

Rheological properties were measured using a rotational rheometer (MCR 302, Anton Paar, GmbH, Graz, Austria) at 25 °C. Steady shear tests were performed over a shear rate range of 0.1–100 s^−1^ and fitted using the power-law model *η* = *K* *γ* ^(^*^n^*^−1)^. Dynamic frequency sweeps were conducted within the linear viscoelastic region (LVE; 1% strain) over an angular frequency range of 0.1–100 rad/s, and G′, G″, and tan δ were recorded.

### 2.14. In Vitro Simulated Digestion

Simulated digestion was performed sequentially through the oral, gastric, and intestinal phases according to the INFOGEST static digestion method. The oral phase was performed at pH 7.0 and 37 °C for 2 min; followed by the gastric phase at pH 3.0 and 37 °C for 2 h; and the intestinal phase at pH 7.0 and 37 °C for 2 h. After digestion, samples were centrifuged at 10,000× *g* and 4 °C for 10 min, and the supernatants were collected for the determination of anthocyanins, total phenolics, DPPH radical-scavenging capacity, and ABTS radical-scavenging capacity. The digestive retention index (RI, %) was calculated as follows: RI = content after digestion/content before digestion × 100 [36,37,38,39,40].

### 2.15. Sensory Evaluation

Ethical approval for the sensory evaluation study was granted by the Science and Technology Ethics Committee of Changchun Normal University (Approval No. 2026018). Sensory evaluation was performed by 12 trained panelists aged 23–28 years, including six men and six women. Four groups were evaluated: CK, Pro-L, Pro, and Pro-H. Samples were coded using three-digit random numbers and presented in randomized order. Panelists rinsed their mouths with warm water between samples. A nine-point scale was used to evaluate color, aroma, astringency harmony, smoothness, and overall acceptability. For astringency harmony, a higher score indicated weaker perceived astringency. All samples were evaluated at least twice [41,42].

### 2.16. Data Processing and Statistical Analysis

All experiments were performed with at least three independent replicates, and results are expressed as the mean ± standard deviation. Data analysis and graphing were performed using IBM SPSsSlalistics 27.0 (IBM Corp. Armionk, NY, USA), Origin Pro 2022b (OriginLab Corporalion Nuitharplon, MA, USA), GraphPad Prsm 9.5.1 (GraphPad Sottware, Uoston, MA, USA), Design-Lxpert 13.0 (Stat-Ease Inc., Minmeapolis, MN, USA), and R sullware 4.3.2 (R Foundalion for Slalislical Corputing.Vienna, Austria). Machine learning and SlIAP analyses were conducted in Python 3.10. 12 (PythonSoftware Foundation, WiImington, DE, USA) using scikit-Icarn 1.3.2, TonsorFlow 2.15.0, SHAP 0.44.1NumPy 1.26.4, pandas 2.1.4, and Matplotib 3.8.2.For comparisons involving more than two treatment levels, one-way analysis of variance (ANOVA) followed by Tukey’s multiple-comparison test was used. For comparisons involving only two groups, Student’s *t*-test was used. Statistical significance was set at *p* < 0.05. Response surface models were established and analyzed using Design-Expert software.

## 3. Results

### 3.1. Modulatory Effect of L-Proline on the Molecular Microenvironment of PC-B2

This study first examined the effect of L-proline on the molecular environment of PC-B2 in a simplified model system to determine whether L-proline could modulate the effective action state of PC-B2 without disrupting the main polyphenol structure. UV-Vis spectroscopy was used to observe the spectral response of the PC-B2-BSA coexisting system, whereas FT-IR spectroscopy was further used to analyze changes in the functional-group environment after the interaction between PC-B2 and L-proline.

**Figure 1:**
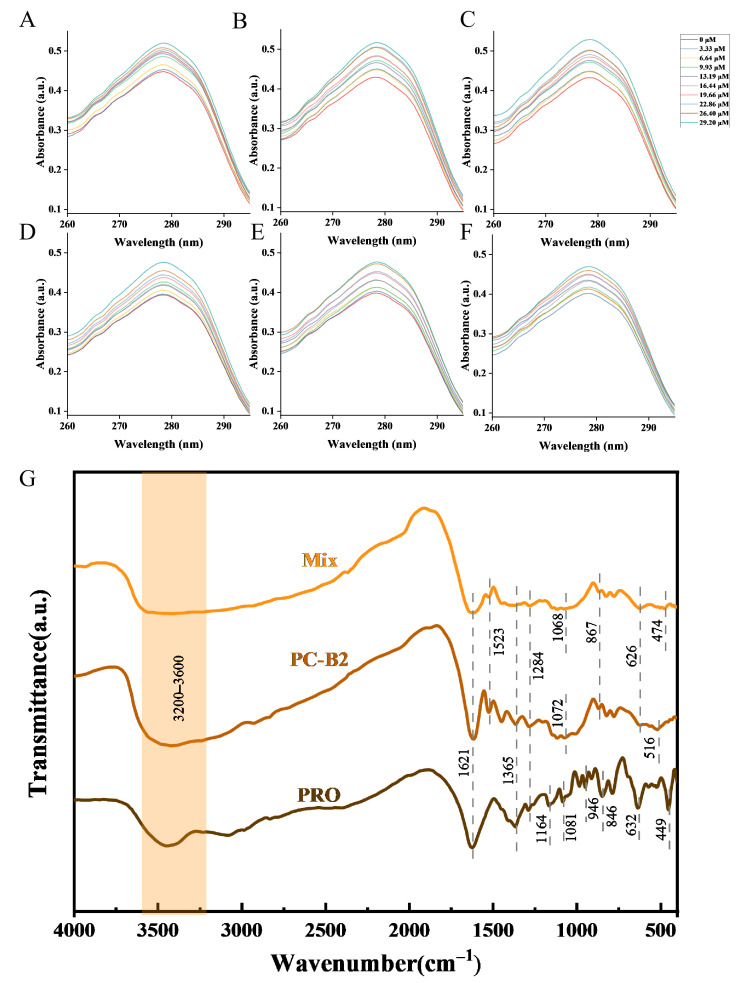
Effects of L-proline on the UV-Vis spectral response of the PC-B2-BSA system and on the functional-group microenvironment of PC-B2. (**A**–**C**): UV-Vis absorption spectra of theBSA-PC-B2 system at 25, 35, and 45 °C with stepwise PC-B2 addition; (**D**–**F**): UV-Vis absorption spectra of the BSA-L-proline-PC-B2 system at 25, 35, and 45 °C with stepwise PC-B2 addition; (**G**): FT-IR spectra of L-proline, PC-B2, and the L-proline-PC-B2 mixed freeze-dried sample. PRO: L-proline; PC-B2: procyanidin B2; Mix: L-proline and PC-B2 mixed freeze-dried sample.

As shown in Figure 1A–C, the CK group exhibited a distinct absorption peak near 280 nm at 25, 35, and 45 °C. This peak is mainly associated with the aromatic structure of PC-B2 and aromatic amino acid residues in BSA. With the gradual addition of PC-B2, the absorbance of the CK group changed regularly at all tested temperatures, indicating that PC-B2 addition altered the spectral response of the BSA-PC-B2 coexisting system. Differences in peak intensity and curve evolution among temperatures further indicated that temperature affected molecular diffusion and non-covalent binding processes in the PC-B2-BSA system.

Based on this observation, Figure 1D–F shows that the Pro group containing L-proline also retained the characteristic absorption near 280 nm at 25, 35, and 45 °C, but its peak intensity, inter-curve spacing, and response trend after PC-B2 addition differed from those of the corresponding CK group. These differences suggest that L-proline did not simply behave as an inert coexisting small molecule; rather, it altered the local spectral environment of the PC-B2-BSA system. Whether this change further affected protein precipitation behavior required subsequent validation using the PC-B2-BSA precipitation assay.

Figure 1G provides further evidence at the functional group level. Compared with pure PC-B2, the freeze-dried L-proline-PC-B2 mixture exhibited peak shifts and spectral deformation in the 3200–3600 cm^−1^ hydrogen-bonding region, near 1621 cm^−1^, and in the low-frequency region around 474 cm^−1^. These changes suggest the occurrence of hydrogen bonding, hydrophobic association, and rearrangement of molecular stacking between L-proline and PC-B2. Meanwhile, the aromatic ring skeleton vibration peak at 1523 cm^−1^ remained in the mixed sample, indicating that the process mainly involved non-covalent microenvironmental reconstruction rather than disruption of the PC-B2 aromatic skeleton.

Therefore, the UV-Vis and FT-IR results jointly support the interpretation that L-proline may modulate the local microenvironment of PC-B2 and may thereby alter its ability to form effective cross-links with proteins. This molecular-level evidence provides a logical basis for subsequent protein precipitation experiments.

### 3.2. Inhibitory Effect of L-Proline on PC-B2-BSA Binding-Induced Precipitation

After confirming that L-proline could modify the local microenvironment of PC-B2, the PC-B2-BSA model was further used to evaluate whether this molecular-environmental change could lead to altered protein precipitation behavior.

**Figure 2:**
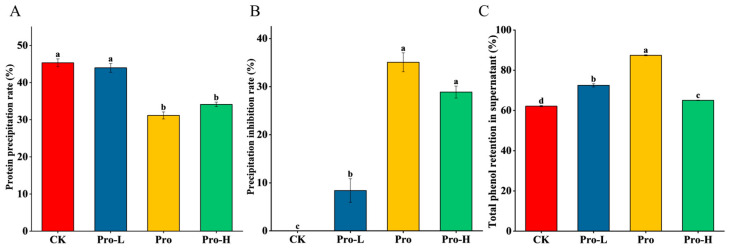
Effects of L-proline on the PC-B2-BSA precipitation system. (**A**): protein precipitation rate; (**B**): precipitation inhibition rate; (**C**): supernatant total phenol retention rate. CK indicates the PC-B2-BSA control system without L-proline addition, and its precipitation inhibition rate was defined as 0%. Pro-L, Pro, and Pro-H correspond to final L-proline concentrations of 0.167, 0.667, and 1.333 mg/mL, respectively. Different lowercase letters above the bars indicate significant differences among groups within the same panel, as analyzed by one-way ANOVA followed by Tukey’s multiple-comparison test (*p* < 0.05).

In the CK group, the protein precipitation rate was 45.3 ± 1.9%, indicating that PC-B2 had strong protein cross-linking and aggregation-inducing capacity. After L-proline addition, the protein precipitation rate showed a dose-related but nonlinear trend. The Pro group, with a final L-proline concentration of 0.667 mg/mL, decreased the protein precipitation rate to 31.2 ± 1.7%, corresponding to a precipitation inhibition rate of 35.1 ± 3.4% (Figure 2A,B). Meanwhile, the soluble total phenol retention rate in the supernatant of the Pro group increased to 87.4 ± 0.4%, which was higher than the 62.0% observed in the CK group (Figure 2C). Together, these findings suggest that an appropriate amount of L-proline reduced the co-sedimentation of PC-B2 with protein complexes and allowed more phenolic substances to remain in the soluble state.

When the L-proline concentration was further increased to the Pro-H level, with a final concentration of 1.333 mg/mL, the precipitation inhibition rate decreased to 28.9 ± 2.2%, and the supernatant total phenol retention rate also decreased compared with the Pro group. This result indicates that the regulatory effect of L-proline on polyphenol–protein interactions did not simply increase with concentration; instead, an appropriate concentration window existed. Excessive L-proline may alter the local polarity or solvation state of the system, thereby weakening its shielding effect against PC-B2-BSA cross-linking.

Thus, L-proline, within an appropriate concentration range, can reduce the capacity of PC-B2 to induce protein precipitation and help maintain some phenolic substances in the soluble state. Because real puree systems contain complex polyphenols, pectin, organic acids, and thermosensitive nutrients, the model system results alone cannot directly determine practical processing performance. Therefore, further validation of the deastringency effect and quality impact in black chokeberry puree was necessary.

### 3.3. Effects of Processing Variables on Deastringency Rate and Nutrient Retention in the Real Puree System

Based on the precipitation inhibition effect observed in the model system, L-proline treatment was further applied to real black chokeberry puree. Because astringency mitigation in the real system may be accompanied by nutrient losses, the deastringency rate was defined according to changes in the capacity of puree supernatants to induce BSA precipitation, and anthocyanin and vitamin C retention rates were evaluated simultaneously.

**Figure 3:**
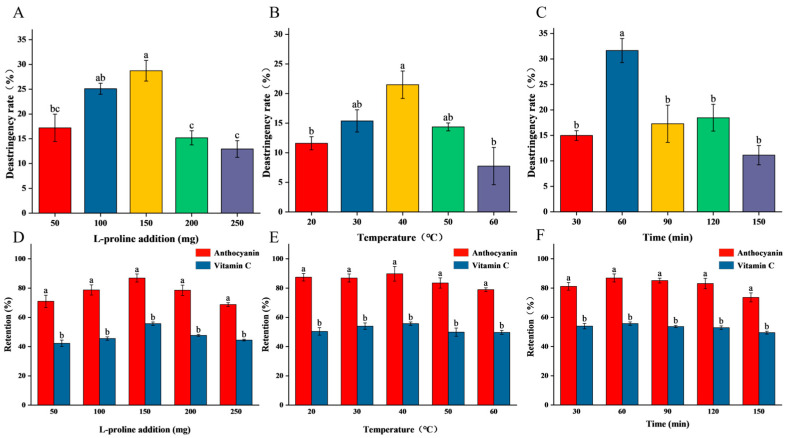
Effects of L-proline dosage, processing temperature, and processing time on the deastringency rate and nutrient retention of black chokeberry puree. (**A**–**C**) show the effects of L-proline dosage, temperature, and time on the deastringency rate, respectively; (**D**–**F**) show changes in anthocyanin and vitamin C retention rates under the corresponding conditions. For panels (**A**–**C**), different lowercase letters indicate significant differences among the different levels of the corresponding single factor, as analyzed by one-way ANOVA followed by Tukey’s multiple-comparison test (*p* < 0.05). For panels (**D**–**F**), comparisons were performed between two groups using Student’s *t*-test (*p* < 0.05). Lowercase letters in panels (**D**–**F**) should be interpreted only within the corresponding two-group comparison and should not be compared with those in panels (**A**–**C**).

Single-factor results showed that the deastringency rate first increased and then tended to plateau with increasing L-proline dosage (Figure 3A). This trend suggests that non-covalent interactions between free amino acids and astringency-related polyphenols such as proanthocyanidins in the puree may have gradually approached saturation. Processing temperature and processing time also had coordinated effects on the deastringency rate. Moderate heating to 40 °C and prolonged treatment time promoted molecular diffusion and the rearrangement of weak interactions, increasing the deastringency rate within a local range (Figure 3B,C).

However, when the temperature increased to 50–60 °C or the processing time exceeded 90 min, the deastringency efficiency decreased, and the retention rates of anthocyanins and vitamin C also declined (Figure 3D–F). This suggests that stronger processing conditions may disrupt the established non-covalent interaction equilibrium and accelerate the loss of thermosensitive nutrients. Vitamin C decreased more markedly under high-temperature and long-duration treatments, indicating a process trade-off between improved deastringency efficiency and nutrient retention.

These single-factor results indicate that L-proline-assisted deastringency has a suitable process window and that single-variable analysis is insufficient to balance deastringency, anthocyanin retention and vitamin C retention. Machine learning-assisted multi-response analysis was therefore used for nonlinear process screening, followed by RSM validation of the recommended process region.

### 3.4. Machine Learning-Assisted Modeling, SHAP Attribution, and Pareto Multi-Objective Optimization

To clarify the integrated effects of L-proline dosage, processing temperature, and processing time on the deastringency rate, anthocyanin retention rate, and vitamin C retention rate, we constructed machine learning models using 125 five-level full-factorial experimental observations. This dataset covered L-proline dosages of 50–250 mg/100 mL, processing temperatures of 20–60 °C, and processing times of 30–150 min, thereby enabling analysis of nonlinear responses and multi-objective trade-offs across a broad process space. Pearson correlation analysis showed that none of the three process variables had strong linear correlations with the three responses. The correlation coefficients of L-proline dosage, temperature, and time with the deastringency rate were 0.14, 0.21, and 0.17, respectively (Figure S1), indicating that nonlinear models were needed to describe this system.

**Figure 4:**
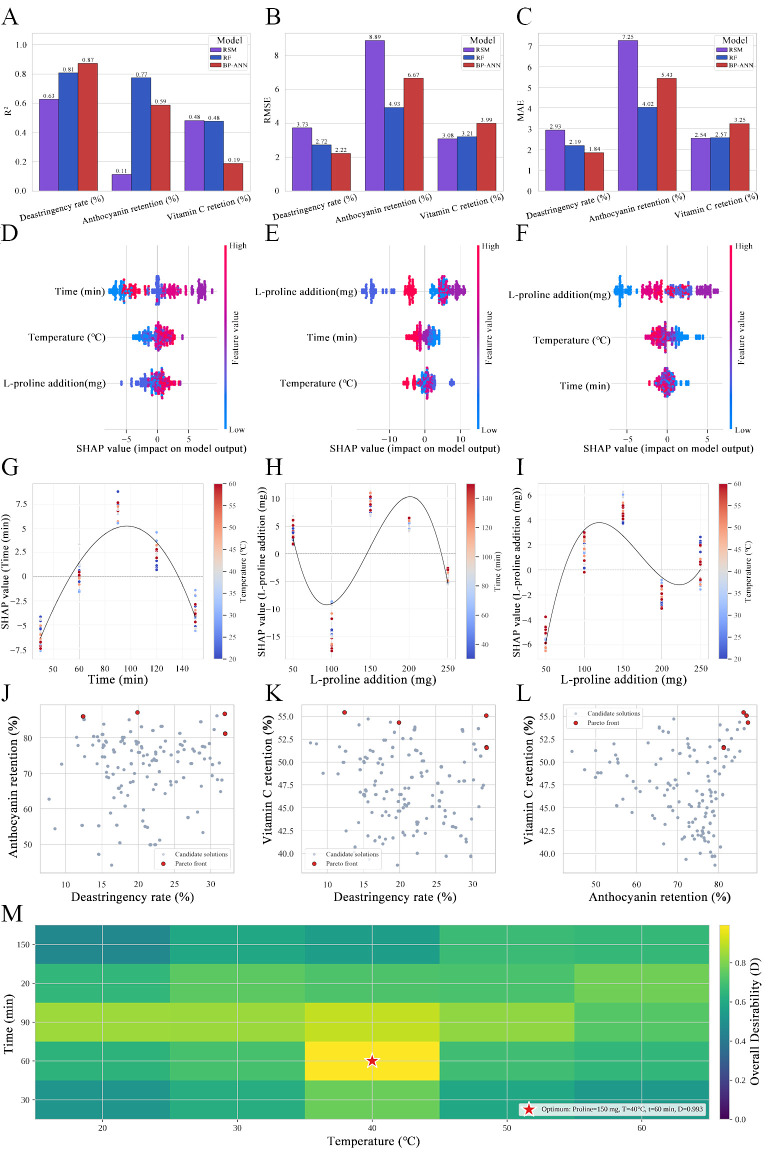
Machine learning-based multi-response modeling, interpretation, and optimization of the L-proline deastringency process. (**A**–**C**): comparison of the predictive performance of the quadratic polynomial response surface model (RSM), random forest (RF) model, and BP artificial neural network model (BP-ANN) for deastringency rate, anthocyanin retention, and vitamin C retention, respectively. Model performance was evaluated using the coefficient of determination (R^2^), root mean square error (RMSE), and mean absolute error (MAE). (**D**–**F**): RF-based SHAP summary plots for deastringency rate, anthocyanin retention, and vitamin C retention, respectively. Each point represents one observation, and the SHAP value indicates the contribution of each process variable to the model output. The color scale represents the relative value of the corresponding feature. (**G**–**I**): SHAP dependence plots showing the nonlinear effects of key variables on the predicted responses, including the effect of processing time on deastringency rate, L-proline dosage on anthocyanin retention, and L-proline dosage on vitamin C retention. (**J**–**L**): Pareto non-dominated solution distributions showing the trade-offs between deastringency rate and anthocyanin retention, deastringency rate and vitamin C retention, and anthocyanin retention and vitamin C retention, respectively. Red points indicate Pareto-front solutions. (**M**): overall desirability heatmap based on RF-predicted multi-response optimization. The star indicates the recommended processing condition, namely 150 mg/100 mL L-proline, 40 °C, and 60 min, with an overall desirability value of 0.993.

Figure 4A–C compares the predictive performance of the quadratic polynomial baseline model, random forest (RF), and BP artificial neural network (BP-ANN). For the deastringency rate, BP-ANN showed the best performance, with a test-set R^2^ of 0.87 and RMSE and MAE values of 2.22 and 1.84, respectively. RF also showed good predictive performance, with a test set R^2^ of 0.81 and RMSE and MAE values of 2.72 and 2.19, respectively. For anthocyanin retention, RF achieved a test set R^2^ of 0.77, which was higher than the 0.59 obtained by BP-ANN, and therefore showed more stable generalization performance. For vitamin C retention, the predictive performance of all models was relatively low; the RF R^2^ was approximately 0.48, suggesting that this response was jointly affected by process conditions and the matrix environment and was therefore more complex. Accordingly, the machine learning prediction for vitamin C retention was used mainly for identifying response trends and supporting multi-response screening, rather than for making a standalone quantitative prediction. The final recommended condition was further evaluated using Pareto analysis, the overall desirability function, Box–Behnken response surface validation, and independent experimental verification to reduce the uncertainty associated with the relatively low vitamin C prediction accuracy. Overall, RF showed the most balanced performance among the three responses, and therefore, it was used as the primary model for subsequent variable interpretation and multi-objective optimization. The predicted-versus-measured relationships and residual distributions of the RF and BP-ANN models are shown in Figures S3 and S4.

The SHAP summary plots based on the RF model showed that the dominant variables differed across responses (Figure 4D–F). Processing time contributed most strongly to the deastringency rate, indicating that the deastringency process required sufficient reaction time. L-proline dosage contributed most to anthocyanin retention and also had a substantial contribution to vitamin C retention. Temperature also affected vitamin C retention, which is consistent with the thermosensitive nature of vitamin C. SHAP dependence plots further revealed nonlinear effects of key variables on the responses (Figure 4G–I). Processing time contributed more strongly to the deastringency rate in the intermediate range, whereas L-proline dosage showed an appropriate range effect on anthocyanin and vitamin C retention. These results indicate that higher L-proline dosage or longer processing time was not necessarily more beneficial; instead, an appropriate processing window existed.

Based on model predictions and variable interpretation, Pareto non-dominated sorting and overall desirability function analysis were subsequently performed using RF-predicted results. Figure 4J–L shows trade-offs among the deastringency rate, anthocyanin retention rate, and vitamin C retention rate; maximizing a single response could not simultaneously achieve both deastringency efficiency and nutrient preservation. Therefore, the overall desirability function was used for balanced multi-objective optimization. The heatmap of the overall desirability function showed that, when the L-proline dosage was fixed at 150 mg/100 mL, regions with higher overall desirability were concentrated at moderate temperature and intermediate processing time, with the highest overall desirability occurring near 40 °C and 60 min (Figure 4M). Considering the Pareto front, SHAP interpretation, and practical process operability, the recommended process was finally determined as 150 mg/100 mL L-proline, 40 °C, and 60 min.

To verify the reliability of this recommended condition, the predicted values from the local quadratic response surface model were compared with independent experimental results (Table 1). Under the recommended condition of 150 mg/100 mL, 40 °C, and 60 min, the measured deastringency rate, anthocyanin retention rate, and vitamin C retention rate were 36.13 ± 1.08%, 88.80 ± 1.47%, and 55.56 ± 1.08%, respectively, which were close to the predicted values of 36.44%, 89.06%, and 56.06%. The relative errors of the three responses were 0.84%, 0.29%, and 0.89%, respectively, and were all below 1%. These results indicate that the machine learning-screened recommended condition showed good model consistency and experimental reproducibility and could be used as the unified treatment parameter for subsequent quality evaluation.

### 3.5. Local Validation of the Recommended Process Region Using the Box–Behnken Response Surface

To further validate the validity of the condition recommended by machine learning within the local process region, a Box–Behnken design was used for quadratic response surface analysis. The BBD ranges were processing time of 30–90 min, processing temperature of 30–50 °C, and L-proline dosage of 100–200 mg/100 mL, which encompassed the condition recommended by machine learning of 150 mg/100 mL, 40 °C, and 60 min. Therefore, this section focuses on local response curvature, factor interactions, and model-fitting reliability near the recommended condition, thereby providing response surface-level complementary validation for the machine learning results.

Table S1 shows that, among the 17 BBD runs, the five center points were all set at 60 min, 40 °C, and 150 mg/100 mL. At the center-point condition, the deastringency rate, anthocyanin retention rate, and vitamin C retention rate were concentrated within 35.8–37.6%, 88.22–90.12%, and 55.7–56.4%, respectively, indicating good experimental repeatability in this region. The ANOVA results further showed that the deastringency model was significant and that the lack-of-fit term was not significant. A, B, C, A^2^, B^2^, and C^2^ were significant, indicating that the deastringency rate was mainly controlled by single-factor effects and quadratic curvature (Table S2). The anthocyanin retention model was also significant with an insignificant lack of fit. C, C^2^, and B × C contributed strongly, indicating that anthocyanin preservation was mainly affected by L-proline dosage and its interaction with temperature (Table S3). The vitamin C retention model also reached significance with an insignificant lack of fit; B, C, AB, AC, BC, and all quadratic terms were significant, suggesting that this thermosensitive index was more sensitive to combined changes in time, temperature, and dosage (Table S4).

**Figure 5:**
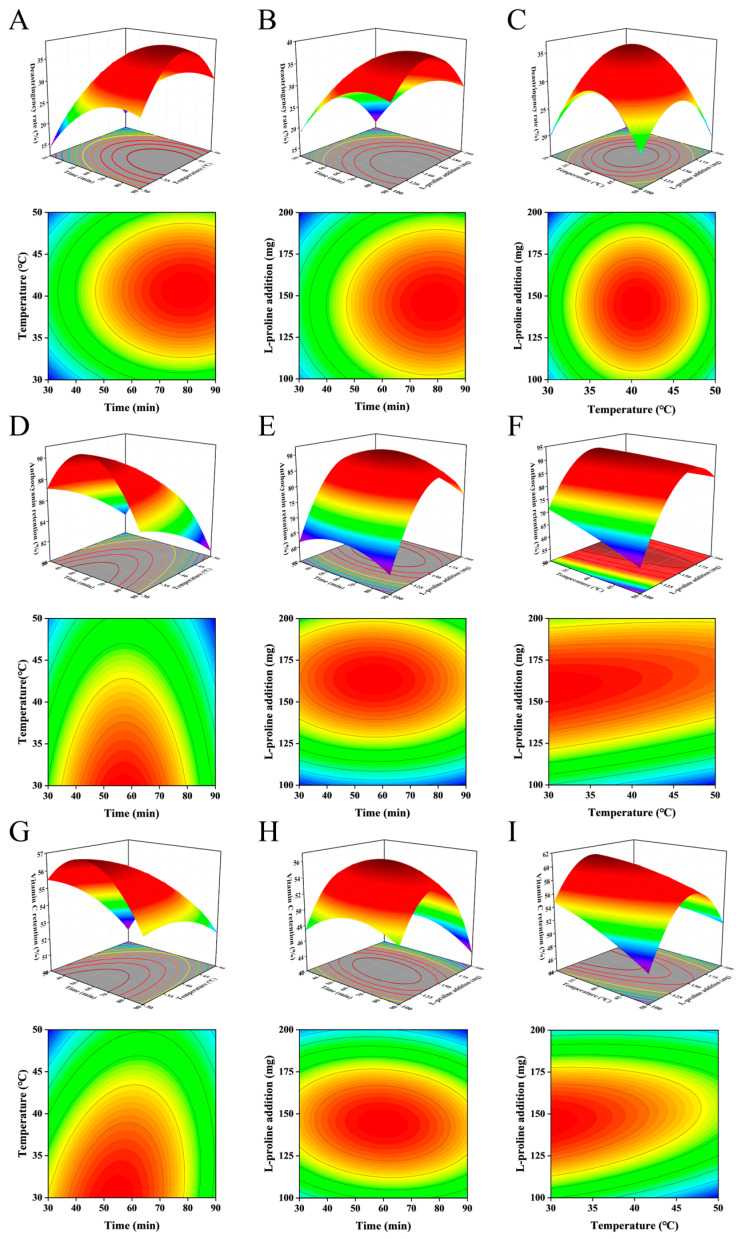
Response surface and contour analysis of the L-proline deastringency process. (**A**–**C**): response surfaces and contour plots for the deastringency rate; (**D**–**F**): response surfaces and contour plots for anthocyanin retention; (**G**–**I**): response surfaces and contour plots for vitamin C retention. The response surface variables were processing time, processing temperature, and L-proline dosage.In the contour plots, warm colors indicate higher predicted response values, whereas cool colors indicate lower predicted response values.

The response surface and contour plots in Figure 5 further show that all three responses had locally favorable regions near the recommended condition. For the deastringency rate (Figure 5A–C), high-value regions were concentrated around moderate processing time, temperature, and L-proline dosage, confirming the existence of an appropriate treatment window. For anthocyanin retention (Figure 5D–F), higher retention regions were mainly concentrated near 150 mg/100 mL and were influenced by the interaction between temperature and dosage. For vitamin C retention (Figure 5G–I), higher temperature and longer processing time were generally unfavorable, but relatively high retention was still maintained near 40 °C, 60 min, and 150 mg/100 mL.

Overall, Tables S1–S4 and Figure 5 indicate that 150 mg/100 mL, 40 °C, and 60 min were located in a local equilibrium region among the deastringency rate, anthocyanin retention rate, and vitamin C retention rate, rather than representing a single-response maximum. This result agrees with the Pareto front and overall desirability function obtained from machine learning, confirming that the recommended condition lies within a local process window characterized by good repeatability, significant model support, and multi-response balance.

### 3.6. Effects of the Optimized L-Proline Treatment on Color and Colloidal Dispersion State

After the recommended process had been determined, it was necessary to assess further whether this non-removal-based deastringency strategy affected the appearance and colloidal stability of the cloudy puree. Because proanthocyanidins and other polyphenolic components were not extensively removed, treatment-induced color deterioration, large-particle flocculation, or charge imbalance would weaken the practical value of this strategy. Therefore, this section evaluated the system state after optimized treatment by considering three aspects: color, particle size distribution, and zeta potential.

**Figure 6:**
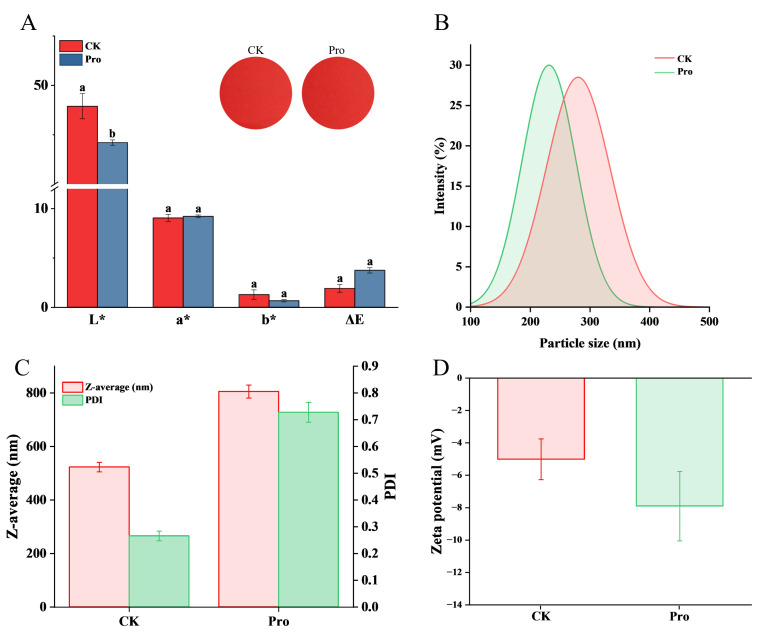
Effects of optimized L-proline treatment on the color and colloidal dispersion state of black chokeberry puree. (**A**): appearance and color difference parameters of different samples, including lightness L*, red–green value a*, yellow–blue value b*, and total color difference ΔE; inset images show representative appearances of CK and Pro samples. (**B**): particle size intensity distribution curves of CK and Pro. (**C**): Z-average and polydispersity index (PDI) of CK and Pro. (**D**): zeta potential of CK and Pro. CK indicates the untreated control; Pro indicates the optimized L-proline treatment group, namely 150 mg/100 mL L-proline, 40 °C, and 60 min. Bar chart data are presented as mean ± standard deviation. Total color difference ΔE was calculated using the untreated control as the reference.Different lowercase letters indicate significant differences between CK and Pro within the same color parameter, as analyzed by Student’s *t*-test (*p* < 0.05).

As shown in Figure 6A, the color parameters of different samples varied to some extent, but all groups retained relatively high L* and a* values, indicating that the black chokeberry puree maintained good lightness and red coloration after treatment. The inset images show that both CK and Pro samples displayed a homogeneous red color, without obvious fading or browning. Compared with CK, the L* and a* values of the Pro group changed slightly, but the red coloration remained prominent. The b* values remained low overall, indicating a limited effect on the yellow tone. Meanwhile, the ΔE value was relatively small, suggesting that the overall color difference induced by optimized L-proline treatment remained within an acceptable range. Thus, deastringency treatment with 150 mg/100 mL L-proline, 40 °C, and 60 min did not noticeably impair the original color characteristics of black chokeberry puree and maintained favorable appearance quality while reducing astringency.

Particle size results further reflected changes in the colloidal structure. Figure 6B shows that the particle size distribution curves of CK and Pro were both unimodal, although the peak positions and distribution ranges shifted to some extent, indicating that L-proline treatment altered the particle population distribution in the puree. The Z-average and PDI results in Figure 6C show that the average hydrodynamic diameter and PDI of Pro increased, suggesting a broader particle-size distribution and moderate restructuring of the system dispersion state. This shift suggests that L-proline treatment did not merely preserve the original particle state but exerted a regulatory effect on the colloidal microstructure of the puree.

Meanwhile, Figure 6D shows that the zeta potential of Pro shifted toward a more negative value compared with CK, indicating increased negative surface charge after treatment. Higher surface charge can enhance electrostatic repulsion among particles, thereby partially offsetting the aggregation tendency associated with particle-size increase and helping to maintain dispersion stability. Therefore, although the optimized treatment modified the particle size distribution and average hydrodynamic diameter, the zeta potential results suggest that obvious colloidal destabilization did not occur.

Overall, optimized L-proline treatment had only a slight effect on the color of black chokeberry puree while moderately restructuring particle distribution, average particle size, and surface charge. The combined color difference and zeta potential results indicate that this process largely maintained the appearance quality and colloidal dispersion stability of the puree while achieving mild deastringency, providing a physical basis for subsequent evaluation of rheological behavior.

### 3.7. Effects of Optimized L-Proline Treatment on the Rheological Properties of the Puree

After confirming that the optimized treatment did not cause obvious color deterioration or colloidal destabilization, its effects on the flow behavior and weak network structure of the puree were further investigated. Rheological properties are directly related to processing transport, filling suitability, and oral texture perception of cloudy purees; therefore, they provide important indicators for evaluating the processing compatibility of this deastringency strategy.

**Figure 7:**
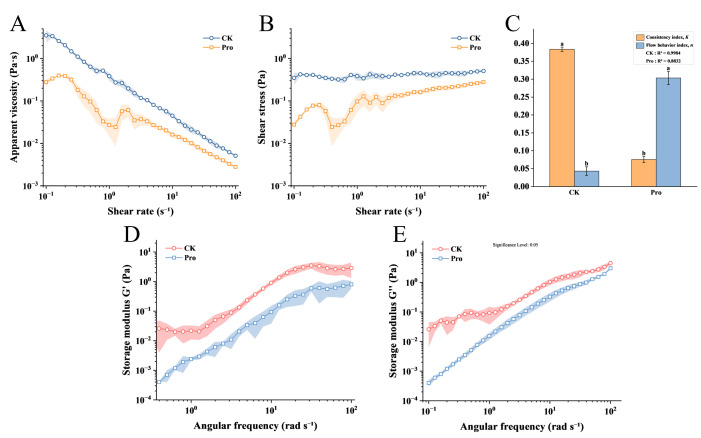
Effects of optimized L-proline treatment on the rheological properties of black chokeberry puree. (**A**): changes in apparent viscosity with shear rate; (**B**): changes in shear stress with shear rate; (**C**): comparison of power-law model fitting parameters, including consistency coefficient *K* and flow behavior index *n*; (**D**): changes in storage modulus G′ with angular frequency; (**E**): changes in loss modulus G″ with angular frequency. CK indicates the untreated control; Pro indicates the optimized L-proline treatment group. The steady shear test range was 0.1–100 s^−1^, and the dynamic frequency sweep range was 0.1–100 rad/s. Shaded regions in curves and error bars in bar charts indicate standard deviations. In panel (**C**), CK and Pro were compared using Student’s *t*-test. Different lowercase letters indicate significant differences between CK and Pro within the same parameter (*p* < 0.05).

As shown in Figure 7A, the apparent viscosity of both CK and Pro decreased markedly with increasing shear rate, indicating typical shear-thinning behavior and confirming that both samples were non-Newtonian pseudoplastic fluids. Compared with CK, Pro showed lower apparent viscosity throughout the shear-rate range, indicating that optimized L-proline treatment reduced the viscous resistance of the puree system. Figure 7B further shows that the shear stress of CK was generally higher than that of Pro, indicating that greater internal structural resistance had to be overcome during flow in the untreated puree; the lower shear stress of Pro suggests weakened interparticle association or a weaker network structure after treatment.

The power-law model fitting results in Figure 7C and Table 2 further support this interpretation. The consistency coefficient *K* of CK was 0.3836 ± 0.0103 Pa·s*^n^*, which was significantly higher than the 0.0755 ± 0.0149 Pa·s*^n^* of Pro, indicating that L-proline treatment substantially reduced the consistency of the puree system. Meanwhile, the flow behavior index *n* increased from 0.0433 ± 0.0206 in CK to 0.3034 ± 0.0315 in Pro, indicating that the degree of shear thinning was weakened after treatment. However, the *n* values of both groups remained below 1, showing that optimized treatment did not change the basic pseudoplastic nature of the puree. The R^2^ values of the power-law fits for CK and Pro were 0.9952 ± 0.0003 and 0.9306 ± 0.0530, respectively, indicating that the model adequately described the steady shear behavior of the samples.

Dynamic frequency sweep results further explained the steady shear changes from the perspective of the viscoelastic structure. Figure 7D shows that G′ of both samples increased with increasing angular frequency, indicating an enhanced elastic response of the particle network under high-frequency disturbance. G′ was generally higher in CK than in Pro, suggesting that the untreated puree had a stronger elastic structure and a stronger interparticle weak network; the lower G′ of Pro indicates that L-proline treatment weakened the elastic network strength. Figure 7E shows that G″ also increased with angular frequency in both samples, indicating clear viscous dissipation under dynamic disturbance. The lower G″ in Pro suggests that the optimized treatment reduced internal friction and viscous resistance.

Taken together, the steady shear and dynamic viscoelastic results show that optimized L-proline treatment significantly reduced apparent viscosity, shear stress, and the consistency coefficient *K* of black chokeberry puree while increasing the flow behavior index *n*, indicating improved flowability and a reduced degree of shear thinning. Meanwhile, G′ and G″ were generally lower in Pro than in CK, suggesting moderate weakening of the weak interparticle network, although the basic viscoelastic characteristics were retained. Therefore, this process reduced the viscous resistance of the puree while preserving the typical rheological attributes of cloudy puree, indicating potential processing applicability.

**Table 2:** Power-law model fitting parameters.

Treatment	*K*/Pa·s*^n^*	*n*	R^2^
CK	0.3836 ± 0.0103 ^a^	0.0433 ± 0.0206 ^b^	0.9952 ± 0.0003 ^a^
Pro	0.0755 ± 0.0149 ^b^	0.3034 ± 0.0315 ^a^	0.9306 ± 0.0530 ^a^

Values are presented as mean ± standard deviation. For each parameter column, different superscript lowercase letters indicate significant differences between CK and Pro, as analyzed by Student’s *t*-test (*p* < 0.05). Superscript letters should be compared only within the same column.

### 3.8. Changes in Bioactive Components and Antioxidant Activity During In Vitro Simulated Digestion

After evaluating processing suitability, this study further examined the effect of optimized treatment on the digestive stability of bioactive components. The functional value of black chokeberry puree depends not only on the content of bioactive compounds at the processing endpoint but also on their retention under gastrointestinal digestion conditions. Therefore, a static in vitro simulated digestion model was used to compare changes in the retention index (RI) of anthocyanins, total phenolics, and antioxidant activity.

**Figure 8:**
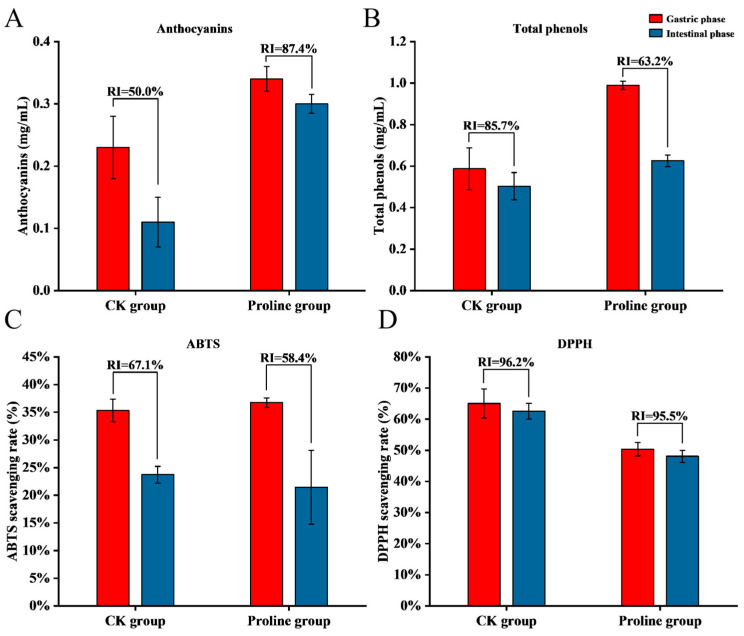
Effects of L-proline deastringency treatment on bioactive components and antioxidant activity of black chokeberry puree during in vitro digestion. (**A**): changes in anthocyanin content; (**B**): changes in total phenol content; (**C**): ABTS radical-scavenging capacity; (**D**): DPPH radical-scavenging capacity. CK: untreated control;

After complete gastrointestinal digestion, the anthocyanin RI of Pro was 87.4%, higher than the 50.0% observed in CK (Figure 8A), and this difference was more pronounced in the intestinal phase. This result indicates that optimized L-proline treatment may improve the apparent stability of anthocyanins during in vitro digestion by altering the polyphenol microenvironment or puree colloidal structure, particularly promoting anthocyanin stability during the higher-pH intestinal phase.

Unlike anthocyanins, the RI of soluble total phenols in Pro was 63.2%, lower than the 85.7% observed in CK (Figure 8B). Because the Folin–Ciocalteu method mainly detects soluble phenolics in the digestion supernatant, this decrease may reflect the transfer of some polymerized polyphenols into insoluble complexes or sedimented phases during digestion rather than complete chemical degradation of total phenolics. Antioxidant activity also showed assay-dependent differences: DPPH radical-scavenging RI was relatively similar between the two groups, whereas ABTS radical-scavenging RI decreased in Pro (Figure 8C,D). This may be related to differences in the solubility states of polyphenol fractions after digestion and to the different sensitivities of the DPPH and ABTS reaction models to sample composition.

Overall, optimized L-proline treatment exerted a pronounced protective effect on anthocyanins during in vitro digestion but had some adverse effects on soluble total phenols and the ABTS response. These results suggest that this strategy has selective effects on the digestive stability of different bioactive components; therefore, its functional significance requires further verification using cell transport or in vivo metabolism studies.

### 3.9. Sensory Quality Evaluation

**Figure 9:**
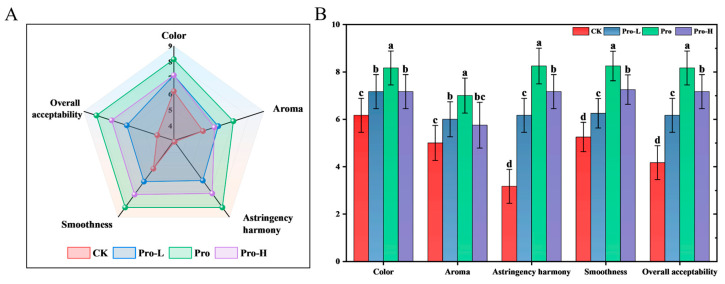
Effects of L-proline deastringency treatment on the sensory quality of black chokeberry puree. (**A**): radar plot; (**B**): bar chart. CK indicates the untreated control; Pro-L indicates the low-dosage L-proline group; Pro indicates the optimized L-proline treatment group; Pro-H indicates the high-dosage L-proline group. Data are presented as mean ± standard deviation. In panel (**B**), different lowercase letters within the same sensory attribute indicate significant differences among treatments, as analyzed by one-way ANOVA followed by Tukey’s multiple-comparison test (*p* < 0.05). Lowercase letters should be compared only within the same sensory attribute and not across different attributes.

Ethical approval for the sensory evaluation study was granted by the Science and Technology Ethics Committee of Changchun Normal University (Approval No. 2026018). After deastringency efficiency, quality preservation, and in vitro digestive stability had been evaluated, sensory evaluation was performed to verify whether these instrumental changes translated into perceptible improvements in mouthfeel. It should be noted that “deastringency rate” and “astringency harmony” are related yet distinct indicators. The deastringency rate is an instrumental parameter calculated from the reduction in BSA protein precipitation capacity, reflecting weakened polyphenol-induced protein aggregation. Thus, it was used to characterize the physicochemical and mechanistic effect of L-proline-mediated astringency mitigation. In contrast, astringency harmony is a sensory score from trained-panel evaluation, with a higher value indicating milder perceived astringency and better mouthfeel balance; therefore, it was used to verify whether the instrumental deastringency effect could be translated into perceptible oral improvement. Although the two indicators showed good correspondence in this study, they cannot be used interchangeably, because sensory astringency is also affected by viscosity, particle perception, acidity, aroma, and oral lubrication. The nine-point scale results showed that the untreated CK group had an astringency harmony score of 3.17 ± 0.72 and an overall acceptability score of 4.17 ± 0.72, indicating obvious sensory defects in the original black chokeberry puree (Figure 9 and Table S5).

The radar plot in Figure 9A shows that CK had the smallest overall profile area, especially in astringency harmony and overall acceptability, which were markedly lower than those of the other treatment groups. This indicates that the original black chokeberry puree had a distinct astringency defect. After L-proline treatment, the profiles of all treated groups expanded outward, and the Pro group showed the largest area, indicating that optimized treatment could improve the overall sensory quality of the puree.

The bar chart in Figure 9B and Table S5 further show that the astringency harmony and overall acceptability scores of CK were 3.17 ± 0.72 and 4.17 ± 0.72, respectively, both at low levels. Scores for the Pro-L group increased compared with CK, indicating that a lower L-proline dosage could already alleviate astringency to some extent. Among all treatments, the recommended Pro group showed the best sensory performance, with significantly higher scores for color, aroma, astringency harmony, smoothness, and overall acceptability than CK (*p* < 0.05). This result indicates that the 36.13% chemical deastringency rate corresponded to obvious sensory improvement, suggesting good agreement between the deastringency effect characterized by reduced BSA protein precipitation capacity and actual astringency mitigation.

## 4. Discussion

### 4.1. Mechanistic Basis for L-Proline Regulation of Proanthocyanidin–Protein Non-Covalent Interactions

Astringency formation is generally associated with non-covalent binding, cross-linked aggregation, and precipitation involving condensed tannins and salivary proteins [42,43,44]. In black chokeberry puree, high levels of proanthocyanidins are both an important source of functional activity and the primary material basis for strong oral astringency [45,46]. Therefore, the deastringency rate does not necessarily depend on tannin removal. A milder strategy is to regulate the effective binding state of proanthocyanidins and thereby weaken their capacity to induce protein precipitation [47,48,49]. Based on this concept, the present study introduced exogenous free L-proline to explore whether it could reduce the tendency of proanthocyanidins to induce protein precipitation and improve the astringency quality of puree by affecting interactions between proanthocyanidins and salivary proteins.

The molecular spectral results showed that L-proline addition altered the UV absorption response of the PC-B2-BSA system near 280 nm (Figure 1A–F). Because absorption at 280 nm is mainly associated with the aromatic structure of PC-B2 and aromatic amino acid residues in proteins, the difference in A_280_ trends between Pro and CK is consistent with, but does not by itself prove, modification of the local spectral environment around the aromatic moieties of PC-B2 and BSA. However, UV–Vis absorption represents a superimposed signal from all chromophores in the measurement system and cannot, on its own, distinguish among site-specific binding, ground state complexation, aggregation-related spectral changes, and non-specific solvation effects; therefore, it should be interpreted together with FT-IR and functional experiments.

The FT-IR results are further consistent with non-covalent microenvironmental regulation involving L-proline and PC-B2 (Figure 1G). Peak shifts or changes in peak shape in the 3200–3600 cm^−1^ hydrogen-bonding region, near 1621 cm^−1^, and in the low-frequency region around 474 cm^−1^ are compatible with changes in hydrogen-bonding environments, hydrophobic-related contacts, and intermolecular packing. However, the assignment of specific interaction modes from conventional KBr-pellet FT-IR spectra alone remains tentative, because this method cannot resolve specific donor–acceptor pairs, binding stoichiometry, or interaction affinity. Meanwhile, the aromatic ring skeleton vibration peak at 1523 cm^−1^ was retained, suggesting that L-proline treatment did not obviously disrupt the basic aromatic skeleton of PC-B2. Therefore, this process can be inferred to be closer to non-covalent microenvironmental reconstruction than to proanthocyanidin degradation or removal.

It is important to delineate the evidential level of each observation. Two findings were directly observed. First, L-proline altered the absorbance evolution of the PC-B2–BSA system at 280 nm at all three tested temperatures (Figure 1A–F). The FT-IR spectrum of the L-proline–PC-B2 mixture also differed from that of pure PC-B2 in the 3200–3600 cm^−1^, 1621 cm^−1^, and 474 cm^−1^ regions, while the 1523 cm^−1^ aromatic skeleton band was retained (Figure 1G). Second, at an appropriate L-proline level, the PC-B2-induced BSA precipitation rate decreased from 45.3% to 31.2%, and soluble total phenolic retention in the supernatant increased from 62.0% to 87.4% (Figure 2). Beyond these observations, the assignment of specific binding modes—hydrogen bonding to phenolic hydroxyls, hydrophobic contact with the pyrrolidine ring, and rearrangement of intermolecular stacking—should be regarded as a working hypothesis. Neither UV–Vis nor diffuse FT-IR can resolve binding site, stoichiometry, or affinity, and the present data do not exclude alternative explanations such as solvation shell perturbation or kinetic stabilization of soluble PC-B2 oligomers. Direct confirmation will require complementary techniques such as isothermal titration calorimetry, steady-state and time-resolved fluorescence quenching of single-Trp BSA, NMR titration, and atomistic molecular dynamics simulation, ideally extended from BSA to authentic salivary proline-rich proteins (PRPs) and mucin MUC5B.

This molecular-level microenvironmental change was supported functionally by the PC-B2-BSA precipitation assay. The protein precipitation rate of the CK group was 45.3%, whereas that of the Pro group decreased to 31.2% after treatment with an appropriate amount of L-proline, with a precipitation inhibition rate of 35.1% (Figure 2A,B). Meanwhile, soluble total phenol retention in the supernatant of the Pro group increased to 87.4% (Figure 2C). These results indicate that an appropriate amount of L-proline reduced the capacity of PC-B2 to form insoluble complexes with BSA, allowing more phenolic compounds to remain in the soluble state. The spectral changes and altered precipitation behavior therefore provide an evidence chain linking microenvironmental alteration to reduced protein-precipitating capacity.

The Pro-H group did not show stronger precipitation inhibition; instead, its inhibitory effect decreased compared with the Pro group, indicating that the regulation of proanthocyanidin–protein interactions by L-proline has an appropriate concentration window rather than a simple dose-dependent relationship [50,51,52]. Excessive free L-proline may alter local polarity, solvation state, or small-molecule competition in the system, thereby weakening its shielding effect against PC-B2-BSA cross-linking. This nonlinear concentration effect also explains why multi-objective process optimization was necessary in the subsequent real puree system [53,54].

Mechanistically, the mode of action of free L-proline should be distinguished from that of salivary proline-rich proteins (PRPs) [55,56]. PRPs are macromolecular proteins containing multiple proline residues, which can provide multivalent binding sites for tannins and participate in precipitation through multipoint cross-linking. In contrast, free L-proline is a low-molecular-weight monomer and does not possess the multivalent binding structure of macromolecular PRPs [57,58,59,60]. Therefore, L-proline in this study is more appropriately understood as a low-molecular-weight microenvironmental modulator. It may reduce protein-precipitating capacity by altering the local aggregation state, solvation shell, and protein contact accessibility of proanthocyanidins. This interpretation still requires further verification using isothermal titration calorimetry, fluorescence quenching, molecular dynamics simulation, or real salivary protein systems.

### 4.2. Multi-Objective Trade-Offs and Model Complementarity in Process Optimization

The actual black chokeberry puree system differs from the simplified PC-B2–BSA model because it simultaneously contains proanthocyanidins, anthocyanins, pectin, proteins, organic acids, vitamin C, and micro- or nanoparticles. Therefore, the effect of L-proline treatment in the actual system depends not only on its regulation of proanthocyanidin–protein interactions but also on the combined effects of temperature, time, and matrix composition. The single-factor results in Figure 3A–C show that L-proline dosage, processing temperature, and processing time all affected the deastringency rate, but their trends were nonlinear. Moderate heating and prolonged treatment time were beneficial for improving the deastringency rate, whereas excessive temperature or overly prolonged treatment could reduce the deastringency effect and accelerate losses of anthocyanins and vitamin C (Figure 3D–F). This indicates a typical processing trade-off: improvements in deastringency efficiency do not always occur in parallel with preservation of nutritional quality.

Based on this characteristic, machine learning models were constructed using 125 five-level full-factorial observations to characterize nonlinear response relationships across a broad process space. The model comparison in Figure 4A–C shows that the predictive abilities of different models differed among response indicators. RF showed relatively balanced performance across the three responses and was therefore more suitable as the primary model for subsequent variable interpretation and multi-objective optimization. The SHAP summary plots in Figure 4D–F further show that different quality targets had different dominant variables: processing time contributed strongly to the deastringency rate, whereas L-proline dosage had a stronger influence on anthocyanin and vitamin C retention. The SHAP dependence plots in Figure 4G–I indicate that key variables exhibited nonlinear interval effects, confirming that L-proline treatment could not be improved simply by increasing dosage, extending time, or elevating temperature; rather, an appropriate processing window existed.

This multi-response trade-off was further reflected in Pareto front and overall desirability function analyses. Figure 4J–L shows a degree of competition among the deastringency rate, anthocyanin retention rate, and vitamin C retention rate. Conditions with high deastringency rate did not necessarily correspond to high nutrient retention, whereas overly mild conditions could not deliver sufficient deastringency. The overall desirability heatmap in Figure 4M shows that, when the L-proline dosage was 150 mg/100 mL, the region near 40 °C and 60 min had a high overall desirability value. Therefore, the condition of 150 mg/100 mL, 40 °C, and 60 min was not a single-response maximum; instead, it represented a balanced compromise among deastringency efficiency, anthocyanin preservation, and vitamin C retention.

In terms of model function, machine learning and Box–Behnken response surface methodology were complementary in this study. The machine learning model was mainly used for nonlinear prediction, variable-contribution analysis, and multi-response balanced screening across a broad process space, whereas the response surface model was used for local curvature analysis and statistical validation around the recommended condition. The response surface and contour plots in Figure 5 show that the deastringency rate, anthocyanin retention rate, and vitamin C retention rate all had locally favorable regions near 150 mg/100 mL, 40 °C, and 60 min, but their high-value regions did not completely overlap. This further confirms that the recommended condition was a multi-response compromise point. The center-point repeatability, model significance, and lack-of-fit results in Tables S1–S4 also support that this condition lies within a relatively stable local process window.

Independent validation further confirmed the reliability of the recommended condition. Table 1 shows that, under the recommended condition of 150 mg/100 mL, 40 °C, and 60 min, the measured deastringency rate, anthocyanin retention rate, and vitamin C retention rate were 36.13%, 88.80%, and 55.56%, respectively, with relative errors below 1% compared with the predicted values from the local quadratic response surface model. This indicates that the machine learning-screened recommended condition had a high overall desirability across the broad process space and was also jointly validated by the local response surface model and independent experiments. Therefore, this condition was appropriate as the unified treatment parameter for subsequent evaluations of color, colloidal stability, rheological properties, in vitro digestion, and sensory quality.

### 4.3. Effects of Non-Removal-Based Deastringency on Colloidal Dispersion and Rheological Behavior of the Puree

The L-proline deastringency strategy proposed in this study is a non-removal-based modulation approach, in which proanthocyanidins and other polyphenolic components were not extensively removed from the puree by filtration, clarification, or precipitation separation. This strategy helps retain polyphenols and color constituents but may also pose risks to colloidal stability. Therefore, determining whether this process is suitable for cloudy puree systems requires a combined evaluation of deastringency effects, color, particle size, zeta potential, and rheological behavior.

The color-difference results show that, after optimized L-proline treatment, the Pro group retained a relatively high a* value and a uniformly red appearance, indicating that the color characteristics of black chokeberry puree were largely maintained (Figure 6A). Although L* and b* changed to some extent, the total color difference ΔE did not indicate substantial deterioration, suggesting that the treatment did not cause obvious fading or macroscopic color instability. Considering that anthocyanins are an important basis for both the color and functional properties of black chokeberry, this result is consistent with the high anthocyanin retention rate shown in Table 1. This indicates that optimized L-proline treatment did not exert a marked adverse effect on the anthocyanin-dominated color system.

Particle size and zeta potential results further show that the optimized treatment did not disrupt the basic dispersion structure of the puree. Figure 6B–D shows that the particle size distributions of both CK and Pro were unimodal. Although the average particle size and PDI of Pro increased, no evidence of large-scale flocculation or particle aggregation was observed. In addition, the zeta potential of Pro shifted toward a more negative value, suggesting enhanced surface charge repulsion among particles, which may help maintain the dispersion stability of the heterogeneous system. It should be noted that zeta potential reflects only the charge-related stability tendency at the processing endpoint and cannot fully represent long-term shelf stability; therefore, accelerated storage, sedimentation rate, and centrifugal stability should be further investigated.

The rheological results showed that the puree retained typical shear-thinning behavior after optimized treatment. In Figure 7A, the apparent viscosity of both CK and Pro decreased with increasing shear rate, indicating that both samples were non-Newtonian pseudoplastic fluids. In Figure 7B, shear stress increased with shear rate, but the shear stress of Pro was generally lower than that of CK, suggesting that optimized L-proline treatment reduced the internal resistance during puree flow. The power-law fitting results in Figure 7C and Table 2 further show that the consistency coefficient K of Pro was significantly lower than that of CK, whereas the flow behavior index n increased significantly. Because the n values of both groups remained below 1, L-proline treatment reduced viscosity and weakened shear-thinning intensity but did not change the basic pseudoplastic nature of the puree. This change may be beneficial for pumping, filling, and drinking flowability, but its effects on perceived thickness and long-term suspension stability require further evaluation through consumer preference and storage tests.

Dynamic frequency sweep results further indicate that L-proline treatment did not fundamentally alter the viscoelastic properties of the puree. Figure 7D,E show that the G′ and G″ values of Pro were generally lower than those of CK, suggesting that L-proline may have attenuated weak interparticle interaction networks; nevertheless, both samples retained frequency-dependent viscoelastic responses. Taken together, Figure 6 and Figure 7 and Table 2 indicate that optimized L-proline treatment improved deastringency indicators while largely maintaining the color, dispersion state, and typical rheological behavior of the cloudy puree, thereby providing physical quality support for its use as a mild processing strategy.

The present study evaluated the colloidal state only at the processing endpoint; therefore, particle size and zeta potential should be interpreted as indicators of initial dispersion stability rather than direct evidence of shelf life stability. The unimodal particle size distribution and more negative zeta potential of the Pro-treated puree suggest potentially acceptable short-term dispersion stability. However, the increased Z-average/PDI and reduced viscosity may increase the risk of slow sedimentation or serum separation during prolonged storage. Future studies should therefore include real-time and accelerated storage tests to monitor sedimentation, serum separation, particle size, zeta potential, rheology, color, anthocyanin retention, vitamin C retention, and microbial stability.

From an industrial perspective, the optimized L-proline dosage was 150 mg/100 mL, which is equivalent to 1.5 g/L. Thus, 1000 L of puree would require approximately 1.5 kg of L-proline. Assuming a food-grade L-proline price of USD 10–30/kg, the direct additive cost would be approximately USD 15–45 per 1000 L of product, excluding auxiliary expenses such as labor, logistics, utilities, and packaging. Because this process uses mild heating and conventional mixing equipment and does not require adsorbents, filtration, or clarification, it has potential scalability. However, pilot-scale production trials, shelf life verification, and a comprehensive techno-economic assessment are still required prior to commercialization.

### 4.4. In Vitro Digestive Stability, Sensory Improvement, and Mechanistic Closure

The functional value of black chokeberry puree depends not only on the retention of bioactive compounds after processing but also on their stability under gastrointestinal conditions. Figure 8 shows that L-proline treatment had selective effects on the in vitro digestive stability of different bioactive components. The digestive retention index of anthocyanins increased from 50.0% in CK to 87.4% in Pro (Figure 8A), indicating that optimized treatment may improve the apparent stability of anthocyanins during simulated gastrointestinal digestion, especially under the higher-pH conditions of the intestinal phase. This result may be associated with L-proline-induced changes in the polyphenol microenvironment, colloidal structural regulation, or the formation of weak interaction networks.

Unlike anthocyanins, the RI of soluble total phenols in Pro was lower than that of CK (Figure 8B), and the ABTS radical-scavenging RI also decreased to some extent (Figure 8C), whereas DPPH RI showed a smaller difference (Figure 8D). This indicates that L-proline treatment did not universally improve the digestive stability of all polyphenol fractions. Because the total phenol assay used in this study mainly detected soluble phenolics in the post-digestion supernatant, the decrease in soluble total phenol RI may indicate that some polymeric polyphenols underwent phase transfer, aggregation, or sedimentation during digestion, rather than simply complete degradation of total phenols. Differences between the DPPH and ABTS results also show that different antioxidant assays have different sensitivities to post-digestion polyphenol composition and solubility state. Therefore, the present results should be described as showing improved apparent retention of anthocyanins during in vitro simulated digestion, but they should not be directly extrapolated to enhanced in vivo bioavailability.

Sensory evaluation further verified the relationship between chemical deastringency indices and perceptible mouthfeel improvement. Figure 9 and Table S5 show that the astringency harmony and overall acceptability scores of CK were 3.17 and 4.17, respectively, indicating obvious sensory defects in the original black chokeberry puree. After optimized treatment, the astringency harmony score of Pro increased to 8.25, and overall acceptability increased to 8.17, demonstrating that L-proline modulation effectively improved perceptible astringency and overall quality. Notably, the overall acceptability of Pro-H was lower than that of Pro, suggesting that excessive L-proline may affect flavor balance or mouthfeel coordination. This is consistent with the decreased precipitation inhibition observed in the high-dosage group in Figure 2.

Sensory improvement corresponded well with reduced BSA protein precipitation capacity, but the two should not be regarded as identical. Astringency perception is not only related to polyphenol-protein precipitation but is also jointly affected by acid-sweet balance, viscosity, particle perception, aroma release, and oral lubrication behavior. Therefore, the sensory results in Figure 9 should be understood as the outcome of multiple interacting factors, among which reduced proanthocyanidin-induced protein precipitation is an important but not exclusive mechanism.

**Figure 10:**
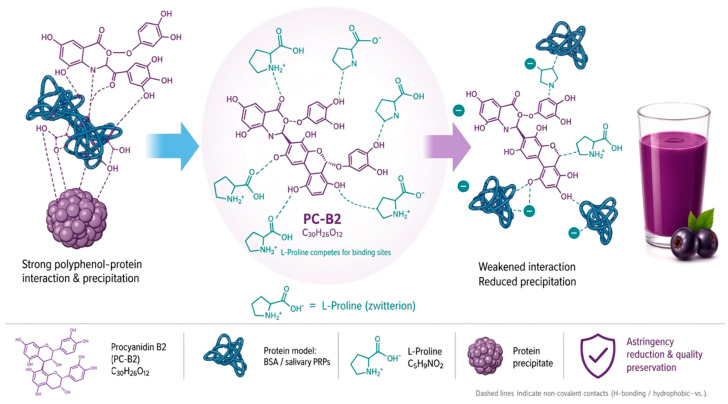
Proposed mechanism of L-proline-mediated deastringency and quality preservation in black chokeberry puree.

Overall, a working model can be constructed from the results of this study. First, L-proline modifies the local microenvironment of PC-B2 through non-covalent interactions (Figure 1). Second, this microenvironmental modification reduces the capacity of PC-B2 to induce BSA precipitation (Figure 2). Third, in the actual puree system, after optimization assisted by RSM and machine learning, appropriate processing conditions achieve a balance among deastringency rate, anthocyanin retention, and vitamin C retention (Figure 3, Figure 4 and Figure 5 and Table 1). Fourth, the optimized treatment largely maintains the color, colloidal dispersion, and rheological characteristics of the puree (Figure 6 and Figure 7 and Table 2). Finally, the treatment results in higher anthocyanin retention during in vitro digestion and significantly improves sensory acceptability (Figure 8 and Figure 9). The mechanism schematic in Figure 10 can be summarized as a continuous action chain involving molecular microenvironmental modulation, reduced protein-precipitating capacity, system quality preservation, and sensory improvement. This model is currently a mechanistic interpretation supported by the available evidence and still requires further validation using authentic salivary systems, oral tribology, and in vivo metabolism studies.

### 4.5. Transferability and Limitations

The transferability of the present findings should be interpreted with caution at three levels. First, at the protein-substrate level, BSA was selected as a model protein because it has a well-characterized globular structure, is commercially available with reproducible purity, and is widely used in polyphenol–protein precipitation studies. This makes it useful for standardized comparison of protein-precipitating capacity. However, BSA is not a structural or functional substitute for human salivary proteins. Salivary proline-rich proteins (PRPs), which are major tannin-binding proteins in vivo, contain proline-rich sequences and conformationally flexible regions that provide multivalent tannin-binding sites. Human saliva also contains mucins, including MUC5B, as well as statherin, histatins, cystatins, enzymes, electrolytes, and other components. These components jointly contribute to salivary lubrication and astringency development. Therefore, the BSA precipitation rate measured in this study should be regarded as a standardized surrogate indicator of proanthocyanidin-induced cross-linking of a globular protein, rather than as a quantitative analogue of PRP- or mucin-mediated precipitation or lubrication changes in real saliva.

Second, at the oral processing level, astringency perception is governed not only by polyphenol–protein association but also by salivary flow, shear, oral lubrication, particle perception, and mucosal friction. These dynamic processes were not reproduced by the static precipitation assay used here. Thus, the BSA-based deastringency rate should be interpreted as a mechanistic indicator rather than a direct measure of perceived astringency. The trained-panel results in Section 3.9 provide sensory-level support for the improved acceptability of the optimized treatment, but they do not fully establish the oral mechanism. Future studies should combine whole human saliva or standardized simulated saliva with oral tribology measurements on saliva-relevant lubricating surfaces and expanded sensory validation.

Third, at the in vivo digestion and bioavailability level, the INFOGEST static digestion model provides useful information on apparent digestive stability, but it does not account for intestinal absorption, first-pass metabolism, or microbial transformation. Accordingly, the higher anthocyanin retention index observed in the Pro-treated group should not be directly interpreted as evidence of enhanced in vivo bioavailability. Further validation using Caco-2 monolayer transport, Ussing chamber experiments, metabolite profiling, or human pharmacokinetic studies is needed to establish physiological relevance.

## 5. Conclusions

This study established a food-grade L-proline-mediated, non-removal strategy for mitigating the astringency of high-tannin *Aronia melanocarpa* puree. Spectroscopic and PC-B2–BSA precipitation data are consistent with a working hypothesis in which L-proline may modify the local microenvironment of procyanidin B2 through non-covalent interactions, thereby attenuating its ability to induce protein precipitation. Direct biophysical confirmation of this hypothesis remains to be obtained. When transferred to the real puree system, multi-response optimization identified 150 mg/100 mL L-proline, 40 °C, and 60 min as the most balanced treatment condition. Under this condition, the deastringency rate reached 36.13%, while anthocyanin retention remained high and key physical attributes, including red color, colloidal dispersion, and shear-thinning rheology, were largely preserved.

The optimized treatment also improved the in vitro digestive retention of anthocyanins and markedly increased sensory scores for astringency balance and overall acceptability. However, the decreases in soluble total phenols and ABTS radical-scavenging retention after digestion indicate that the protective effect was selective among phenolic fractions. Therefore, L-proline treatment should be regarded as a mild astringency modulation approach that balances sensory improvement and quality preservation, rather than as a universal enhancer of polyphenol stability or bioavailability. Further studies using real saliva systems, oral tribology, storage trials, and in vivo or cellular digestion–absorption models are needed to clarify the complete mechanism and practical shelf life performance.

## Figures and Tables

**Table 1:** Comparison between model-predicted and experimentally measured values under the optimal conditions.

Response	Predicted Value/%	Measured Value/%	Relative Error/%
Deastringency rate	36.44	36.13 ± 1.08	0.84
Anthocyanin retention rate	89.06	88.80 ± 1.47	0.29
Vitamin C retention rate	56.06	55.56 ± 1.08	0.89

## Data Availability

The original contributions presented in this study are included in the article and Supplementary Materials. Further inquiries can be directed to the corresponding author.

## Supplementary Materials

The following supporting information can be downloaded at: https://www.mdpi.com/article/10.3390/foods15132388/s1, Table S1: Box–Behnken experimental design and response values; Table S2: ANOVA for the quadratic regression model of deastringency rate; Table S3: ANOVA for the quadratic regression model of anthocyanin retention; Table S4: ANOVA for the quadratic regression model of vitamin C retention; Table S5: Sensory scores under different treatments; Figure S1: Pearson correlation heatmap between process variables and response values; Figure S2: Prediction accuracy and residual analysis of the random forest model; Figure S3: Prediction performance and residual analysis of the BP-ANN model; Figure S4: Feature importance analysis of the random forest model.

## Abbreviations

The following abbreviations are used in this manuscript:

ABTS	2,2′-azino-bis(3-ethylbenzothiazoline-6-sulfonic acid)
ANOVA	analysis of variance
a.u.	arbitrary units
BBD	Box–Behnken design
BCA	bicinchoninic acid
BP-ANN	back-propagation artificial neural network
BSA	bovine serum albumin
CK	control group
DPPH	2,2-diphenyl-1-picrylhydrazyl
FT-IR	Fourier-transform infrared spectroscopy
INFOGEST	standardized static in vitro gastrointestinal digestion protocol
LVE	linear viscoelastic region
MAE	mean absolute error
Mix	L-proline and PC-B2 mixed freeze-dried sample
MW	molecular weight
PBS	phosphate-buffered saline
PC-B2	procyanidin B2
PC-B2-BSA	procyanidin B2-bovine serum albumin model system
PDI	polydispersity index
PRPs	proline-rich proteins
Pro	L-proline-treated group; in optimized puree experiments, the optimized L-proline treatment group
Pro-L	low-dose L-proline group
Pro-H	high-dose L-proline group
R^2^	coefficient of determination
RF	random forest
RI	digestive retention index
RMSE	root mean square error
RSM	response surface methodology
SHAP	Shapley additive explanations
SPSS	Statistical Package for the Social Sciences
TAC	total anthocyanin content
UV-Vis	ultraviolet–visible spectroscopy
