# Supplementary material for: L-Proline-Mediated Modulation of Astringency in Black Chokeberry Puree: Molecular Interactions, Process Optimization, and Quality Preservation
Source: Foods. 2026 Jul 4;15(13):2388. doi: 10.3390/foods15132388 (PMC13361080; doi:10.3390/foods15132388)
Supplement: Supplementary file 1 [file foods-15-02388-s001.zip › foods-4379457-supplementary.pdf]

Table S1. Box-Behnken experimental design and response values

No.	A: Time (min)	B: Temp. (°C)	C: Pro (mg)	Y_1: Deastringency (%)	Y_2: Anthocyanin retention (%)	Y_3: Vitamin C retention (%)
1	30	30	150	13.2	86.55	55.8
2	90	30	150	27.7	85.33	53.6
3	30	50	150	15.1	82.11	50.9
4	90	50	150	30.5	81.12	51.8
5	30	40	100	20.1	61.23	46.8
6	90	40	100	31.3	59.89	48.5
7	30	40	200	14.3	79.71	45.8
8	90	40	200	27.4	76.74	44.3
9	60	30	100	18.7	72.12	52.7
10	60	50	100	20.8	59.68	47.2
11	60	30	200	17.2	80.12	46.0
12	60	50	200	19.7	81.50	45.9
13	60	40	150	35.9	90.12	55.7
14	60	40	150	36.3	88.66	56.1
15	60	40	150	37.6	89.20	56.4
16	60	40	150	35.8	89.12	55.9
17	60	40	150	36.6	88.22	56.2

Note: A, B, and C represent treatment time, treatment temperature, and L-proline addition, respectively; Y_1, Y_2, and Y_3 represent deastringency rate, anthocyanin retention, and vitamin C retention, respectively. All response values are measured values.

Table S2. ANOVA for the quadratic regression model of deastringency rate

Source	Sum of squares	df	Mean square	F value	p value
Model	1238.90	9	137.66	78.25	<0.0001
A-Time	367.21	1	367.21	208.73	<0.0001
B-Temperature	10.81	1	10.81	6.15	0.0423
C-Pro	18.91	1	18.91	10.75	0.0135
AB	0.2025	1	0.2025	0.1151	0.7444
AC	0.9025	1	0.9025	0.5130	0.4970
BC	0.0400	1	0.0400	0.0227	0.8844
A^2	119.17	1	119.17	67.74	<0.0001
B^2	379.60	1	379.60	215.78	<0.0001
C^2	259.13	1	259.13	147.30	<0.0001
Residual	12.31	7	1.76	—	—
Lack of fit	10.22	3	3.41	6.52	0.0509 (not significant)
Pure error	2.09	4	0.5230	—	—

Note: A, B, and C represent treatment time, treatment temperature, and L-proline addition, respectively; AB, AC, and BC denote interaction terms; A^2, B^2, and C^2 denote quadratic terms. $p < 0.05$ indicates statistical significance; $p < 0.01$ indicates high statistical significance. The nonsignificant lack-of-fit term indicates good model fit.

Table S3. ANOVA for the quadratic regression model of anthocyanin retention

Source	Sum of squares	df	Mean square	F value	p value
Model	1723.09	9	191.45	189.17	<0.0001
A-Time	5.31	1	5.31	5.25	0.0557
B-Temperature	48.56	1	48.56	47.98	0.0002
C-Pro	530.57	1	530.57	524.23	<0.0001
AB	0.0132	1	0.0132	0.0131	0.9122
AC	0.6642	1	0.6642	0.6563	0.4445
BC	47.75	1	47.75	47.18	0.0002
A^2	90.05	1	90.05	88.97	<0.0001
B^2	1.85	1	1.85	1.82	0.2190
C^2	953.31	1	953.31	941.93	<0.0001
Residual	7.08	7	1.01	—	—
Lack of fit	5.07	3	1.69	3.36	0.1361 (not significant)

Note: A, B, and C represent treatment time, treatment temperature, and L-proline addition, respectively; AB, AC, and BC denote interaction terms; A^2, B^2, and C^2 denote quadratic terms. $p < 0.05$ indicates statistical significance; $p < 0.01$ indicates high statistical significance. The nonsignificant lack-of-fit term indicates that the model adequately describes changes in anthocyanin retention.

Table S4. ANOVA for the quadratic regression model of vitamin C retention

Source	Sum of squares	df	Mean square	F value	p value
Model	320.82	9	35.65	146.39	<0.0001
A-Time	0.1513	1	0.1513	0.6211	0.4565
B-Temperature	18.91	1	18.91	77.66	<0.0001
C-Pro	21.78	1	21.78	89.45	<0.0001
AB	2.40	1	2.40	9.87	0.0164
AC	2.56	1	2.56	10.51	0.0142
BC	7.29	1	7.29	29.94	0.0009
A^2	22.61	1	22.61	92.87	<0.0001
B^2	2.17	1	2.17	8.90	0.0204
C^2	230.10	1	230.10	944.97	<0.0001
Residual	1.70	7	0.2435	—	—
Lack of fit	1.41	3	0.4708	6.45	0.0518 (not significant)

Note: A, B, and C represent treatment time, treatment temperature, and L-proline addition, respectively; AB, AC, and BC denote interaction terms; A^2, B^2, and C^2 denote quadratic terms. $p < 0.05$ indicates statistical significance; $p < 0.01$ indicates high statistical significance. The nonsignificant lack-of-fit term indicates reliable model fitting.

Table S5. Sensory scores under different treatments

Treatment group	Color	Aroma	Astringency coordination	Fineness	Overall acceptability
CK	6.17 ± 0.72^{c}	5.00 ± 0.74^{c}	3.17 ± 0.72^{d}	5.25 ± 0.62^{d}	4.17 ± 0.72^{d}
Pro-L	7.17 ± 0.72^{b}	6.00 ± 0.74^{b}	6.17 ± 0.72^{c}	6.25 ± 0.62^{c}	6.17 ± 0.72^{c}
Pro	8.17 ± 0.72^{a}	7.00 ± 0.74^{a}	8.25 ± 0.75^{a}	8.25 ± 0.62^{a}	8.17 ± 0.72^{a}
Pro-H	7.17 ± 0.72^{b}	5.75 ± 0.97^{b}	7.17 ± 0.72^{b}	7.25 ± 0.62^{b}	7.17 ± 0.72^{b}

Note: A 9-point scale was used; a higher astringency coordination score indicates weaker astringency and a more coordinated mouthfeel. Data are expressed as mean ± standard deviation; different lowercase letters in the same column indicate significant differences ($p < 0.05$). CK, Pro-L, Pro, and Pro-H represent the control group, low-level L-proline treatment group, optimized L-proline treatment group, and high-level L-proline treatment group, respectively.

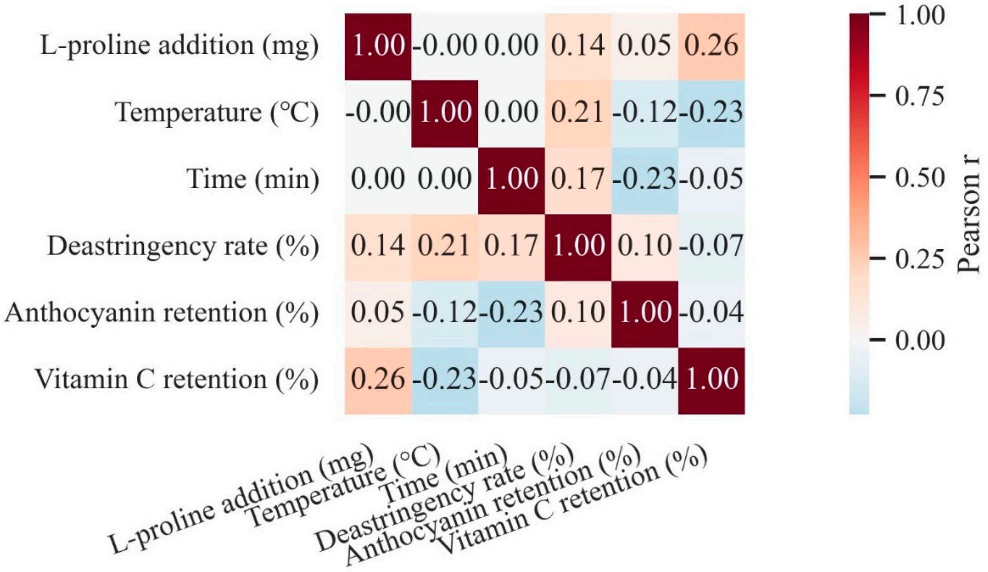

Figure S1. Pearson correlation heatmap between process variables and response values.

Note: Variables include L-proline addition, treatment temperature, treatment time, deastringency rate, anthocyanin retention, and vitamin C retention. Colors indicate Pearson correlation coefficients.

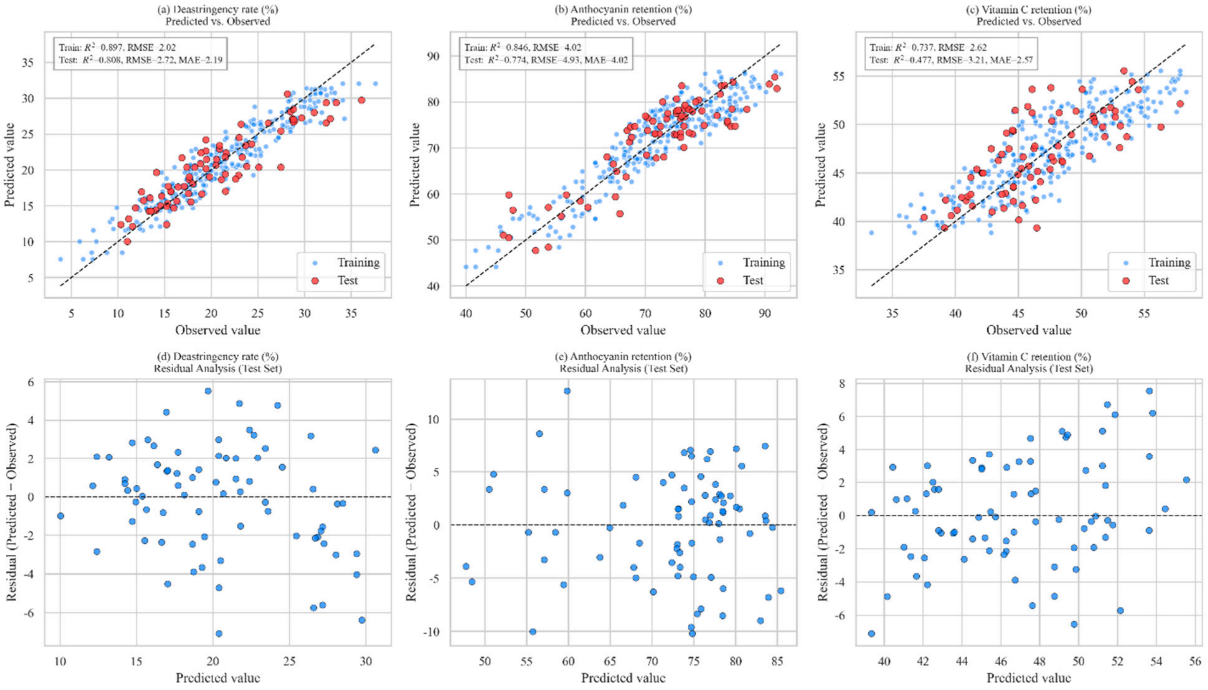

Figure S2. Prediction accuracy and residual analysis of the random forest model.

Note: (a-c) Predicted versus observed values for deastringency rate, anthocyanin retention, and vitamin C retention, respectively; (d-f) residual plots for deastringency rate, anthocyanin retention, and vitamin C retention, respectively. The dashed line indicates the 1:1 reference line.

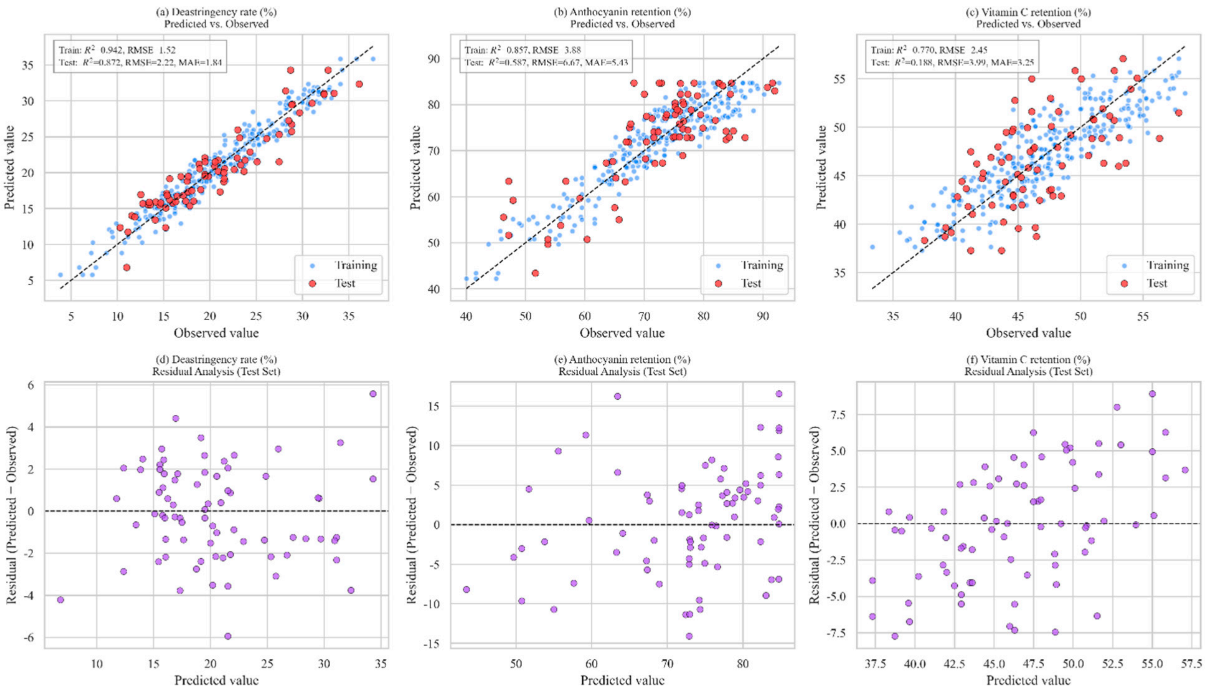

Figure S3. Prediction performance and residual analysis of the BP-ANN model.

Note: (a-c) Predicted versus observed values for deastringency rate, anthocyanin retention, and vitamin C retention, respectively; (d-f) residual plots for deastringency rate, anthocyanin retention, and vitamin C retention, respectively. The dashed line indicates the 1:1 reference line.

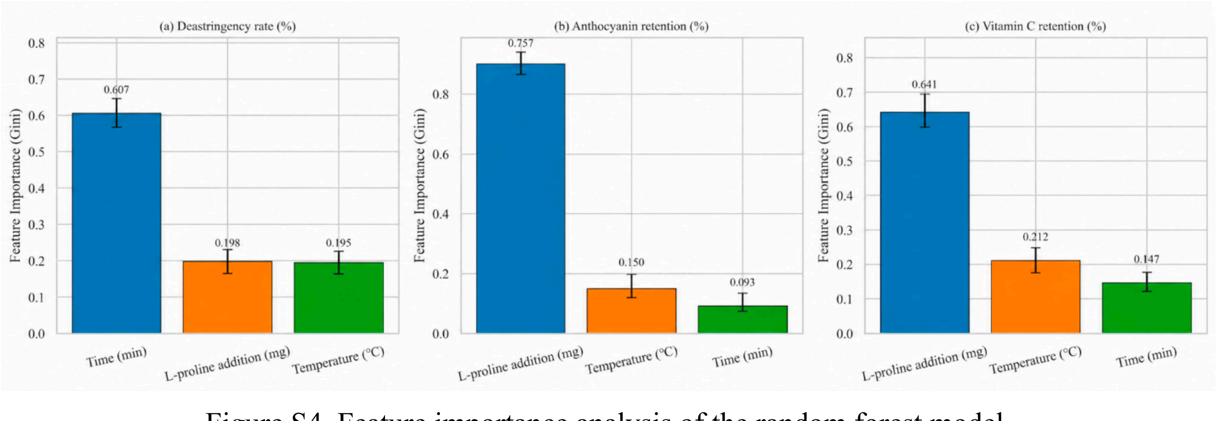

Figure S4. Feature importance analysis of the random forest model.

Note: (a) deastringency rate; (b) anthocyanin retention; (c) vitamin C retention. Bars indicate Gini-index-based feature importance, and error bars indicate variation across repeated model runs.
